# Anaerobic metabolism promotes breast cancer survival via Histone-3 Lysine-18 lactylation mediating PPARD axis

**DOI:** 10.1038/s41420-025-02334-x

**Published:** 2025-02-08

**Authors:** Ying Xu, Weiwei Meng, Yingqi Dai, Lin Xu, Ning Ding, Jinqing Zhang, Xuewei Zhuang

**Affiliations:** 1 Department of Thyroid and Breast Surgery, Shandong Provincial Third Hospital, Shandong University, Jinan, China; 2 Department of Clinical Laboratory, Shandong Provincial Third Hospital, Shandong University, Jinan, China; 3 Translational Medicine Laboratory, Shandong Provincial Third Hospital, Shandong University, Jinan, China; 4 Department of Anesthesiology, Shandong Provincial Key Medical and Health Laboratory of Intensive Care Rehabilitation, Shandong Provincial Third Hospital, Cheeloo College of Medicine, Shandong University, Jinan, China

**Keywords:** Cancer metabolism, Cell signalling

## Abstract

Histone lactylation plays a crucial role in cancer progression, but its impact on breast cancer (BC) tumorigenesis is still unclear. We utilized chromatin immunoprecipitation sequencing with H3K18la antibodies, transcriptomics of clinical BC samples, and proteomics and ATAC-seq analyses of in vivo tumors to identify the genes regulated by H3K18la and the transcription factor PPARD. qPCR and Western blot assays were used to detect expressions of molecules. We discovered that H3K18la levels were higher in BC tissues compared to adjacent non-cancerous tissues. H3K18la promoted the expression of PPARD, which in turn influenced the transcription of AKT, but not ILK. ATAC-seq analysis revealed that glycolysis in BC cells enhanced chromatin accessibility. Additionally, we confirmed that HDAC2 and HDAC3 act as “erasers” for H3 lysine lactylation. During the proteomics analysis, AKT-phosphorylation in the aerobic respiration inhibitor group exhibited an apparent disparity and activity. Our study demonstrated that changes in H3K18la in BC and its downstream transcription factor PPARD support cell survival under anaerobic glycolysis conditions. PPARD accelerated cancer proliferation by promoting the transcription and phosphorylation of AKT. This highlights the therapeutic potential of targeting the H3K18la/PPARD/AKT axis in breast cancer, providing new insights into epigenetic regulation and cancer metabolism (Trial registration: The study was approved by the Research Ethics Committee Shandong Provincial Third Hospital (KYLL-2023057; https://www.medicalresearch.org.cn/)).

## Background

According to statistics from the National Cancer Center of China, breast cancer (BC) has become the second most common malignant tumor among women [1]. Although its mortality rate is lower than that of lung, liver, and stomach cancers, it remains a significant concern. Efforts to reduce the mortality rate of breast cancer require multifaceted approaches and continue to be a substantial challenge. The fundamental approach to this goal is to exhaustively understand the molecular mechanism of tumorigenesis and malignant progression of breast cancer [2].

Lactate, traditionally viewed as a mere by-product of anaerobic glycolysis, is now recognized as a versatile signaling molecule with significant roles in autocrine, paracrine, and endocrine systems [3]. Under aerobic conditions, glucose and glycogen metabolism in both experimental animals and humans predominantly leads to lactate production rather than pyruvate. Lactate’s role extends beyond metabolism; it can be converted into lactyl-CoA within cells [4]. The recent discovery of histone lactylation by Zhang et al. in 2019 underscores its role as a novel epigenetic modification influenced by intracellular lactate metabolism [5]. This modification regulates gene transcription and expression, which is particularly relevant in the context of tumor cells that predominantly utilize glycolysis for energy production even in the presence of oxygen, known as the Warburg effect [6–8]. This metabolic pathway results in higher lactate production in tumor cells compared to normal cells, making histone lactylation a critical area of study in cancer research. Histone lactylation has been shown to influence tumor metabolism, angiogenesis, and immunosuppression [5, 9, 10]. Targeting specific lactylation sites presents a promising strategy for cancer therapy. Enzymes involved in lactate metabolism, such as lactate dehydrogenase (LDH) and monocarboxylate transporters (MCTs), are potential therapeutic targets. Furthermore, the interplay between lactate, histone lactylation, and gene expression suggests new avenues for therapeutic intervention in breast cancer treatment by modulating these epigenetic modifications.

**Table 1:** Comparisons of H3K18la expression in breast cancer tissues in distinct ages and TNM stages.

Characteristics	Number	H3K18la expression	95% Confidence interval	*P*-value
*Age, years*				<0.0001
<45	45	45.464 ± 10.172	−16.26 to −7.222	
≥45	31	33.721 ± 8.996		
*TNM stage*				
T1	29	30.519 ± 8.322		
T2	25	34.286 ± 7.218	−0.5221 to –8.056^a^	0.0839
T3	16	42.722 ± 7.724	7.104–17.30^a^ 3.633–13.24^b^	<0.0001^a^ 0.001^b^
T4	6	50.366 ± 8.937	12.17–27.53^a^ 9.067–23.09^b^ −0.3890 to –15.68^c^	<0.0001^a^ <0.0001^b^ 0.0610^c^
N1	40	31.975 ± 6.274		
N2	26	36.591 ± 7.962	1.102–8.130^d^	0.0108^d^
N3	10	47.015 ± 8.136	10.30–19.78^d^ 4.368–16.48^e^	<0.0001^d^ 0.0013^e^
M0	72	40.388 ± 10.022		
M1	4	51.143 ± 4.121	0.6710–20.84	0.0369

^a^T1 versus T2–T4.

^b^T2 versus T3 and T4.

^c^T3 versus T4.

^d^N1 versus N2 and N3

^e^N2 versus N3.

**Table 2:** Different expression proteins in DCA and ROT groups of breast cancer subcutaneous tumors of nude mice by proteomics sequence analysis.

Compare_filtered	Sig_down	Sig_up	All
Rotenote_VS_Control	366	385	751
DCA_VS_Control	343	275	618

Sig: significant.

Peroxisome proliferator-activated receptor delta (PPARD) is a ligand-dependent nuclear transcription factor integral to regulating glucose and lipid metabolism, cell proliferation, differentiation, and inflammation [11]. Synthetic ligands like GW501516 and GW0742 activate PPARD [12], shifting the body’s fuel preference from glucose to lipids and enhancing muscle endurance, suggesting potential therapeutic applications for obesity and dyslipidemia. However, these ligands also exhibit tumor-promoting effects in preclinical models, raising safety concerns. PPARD is overexpressed in various cancers, including breast, colon, lung, and head and neck cancers, and is regulated by oncogenic pathways such as k-Ras, Wnt, and Src [13–17]. Its disruption can suppress tumor development and metastasis. In lung cancer, PPARD overexpression is linked to poor prognosis, though its role remains ambiguous, with studies indicating both tumor-promoting and inhibitory effects [13]. In gastric cancer, PPARD overexpression in genetically engineered mice induces invasive adenocarcinoma and chronic inflammation, highlighting its therapeutic potential but necessitating further investigation into its role in metabolism and tumor microenvironment modulation [15]. PPARD’s significant regulatory functions make it a promising yet complex target for therapeutic intervention in cancer and metabolic disorders. In our study, we clustered the crucial factor- PPARD from ChIP-seq analysis of H3K18la antibody in BC tissues compared to the para-cancerous tissues. However, whether the PPARD participates in the tumorigenesis of BC survival via impacting the transcript process of certain downstream genes is still unknown.

In addition, glycolysis metabolism-mediated histone lactylation was discovered in colorectal cancer, hepatocellular carcinoma, and ovarian cancer [18–20], whereas rare evidence was found with abnormal respiration metabolism in BC. To find out the above unspecified mechanism, we constructed multiple in vitro experiments and in vivo animal models to substantiate glycolysis-mediated H3K18la downstream transcript factors and their potential molecular mechanism as well as signaling pathways for sustaining cancer cells survival.

## Results

### Glycolysis promoted breast cancer cell survival

**Fig. 1:**
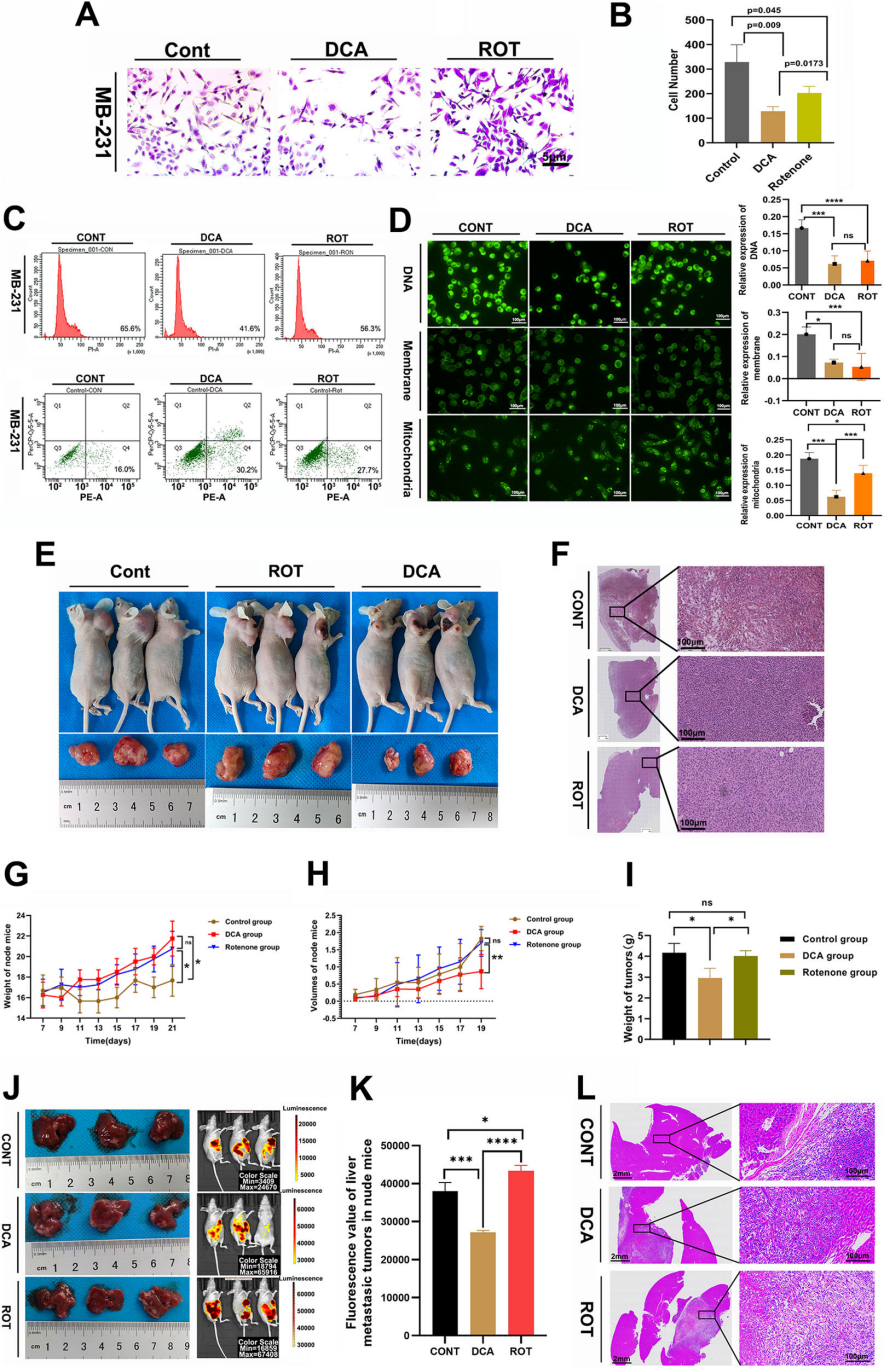
Anaerobic respiration metabolism promoted breast cancer cell proliferation in vitro and in vivo experiments. **A** and **B** MB-231 cell proliferation increased in the ROT group compared to the DCA group. Scale bars: 5 and 10 μm. **C** Flow cytometry assay showed cell cycle and cell apoptosis changes in three groups. **D** Changes in dual-chain DNA, cell membrane, and mitochondria of MB-231 cells treated with PBS, DCA, and Rotenone for 24 h. **E** Subcutaneous breast cancer (4T1) tumors in mice treated with DCA and Rotenone for 21 days (*n* = 3). **F** Hematoxylin and Eosin staining showed tumor intensity differences among the three groups. **G**–**I** Weight curves, volume curves, and weight of subcutaneous tumors of nude mice in three groups (*n* = 3). **J** Breast cancer (4T1-L) liver metastasis models treated with DCA and Rotenone compared to control. **K** In vivo fluorescence imaging detections were performed per 3 days. **L** Hematoxylin and Eosin staining showed differences in liver tumor intensity. All experiments had three biology repetitions with similar results. Data are mean ± SD. *P < 0.05, **P ≤ 0.01, ***P < 0.001, ****P ≤ 0.0001.

To investigate the impact of the Warburg effect on cancer cell proliferation, we established in vitro experimental groups using dichloroacetic acid (DCA), an inhibitor of anaerobic respiration, and Rotenone, an inhibitor of aerobic respiration. Cell monoclonal proliferation assays revealed that the number of MB-231 cells increased under the glycolysis condition with ROT interference compared to the DCA and control groups (Fig. 1A, B). These findings were further corroborated by flow cytometry assays, which showed cell cycles and apoptosis outcomes (Fig. 1C), indicating that the ROT group under hypoxia showed significantly reduced apoptosis compared to the other two groups. In contrast, the DCA group exhibited increased apoptosis, further demonstrating the vulnerability of cancer cells when anaerobic metabolism is disrupted. To explore whether abnormal respiratory metabolism influenced cancer cell apoptosis, we examined alterations in mitochondrial structure, double-stranded DNA, and cell membranes following 24 h of treatment with DCA and Rotenone (Fig. 1D). Apoptosis was first observed in mitochondria. Notably, membrane and DNA damage were not significantly different at 24 h in the DCA and ROT groups compared to the control. However, a significant difference in mitochondrial apoptosis was observed between the two drug treatments, with cells treated with DCA showing more pronounced apoptotic features. Furthermore, in vivo experiments were performed by establishing subcutaneous tumors in nude mice using 4T1 breast cancer cells. Tumor growth in the ROT group remained robust, with tumor sizes comparable to those in the control group, indicating that inhibition of aerobic respiration did not hinder tumor proliferation. In contrast, treatment with the anaerobic respiration inhibitor-DCA, resulted in a significant reduction in tumor growth compared to the control and ROT groups (*p* < 0.05; Fig. 1E). The Rotenone-treated tumors displayed a higher proportion of malignant cells with dense cellularity compared to the other groups (Fig. 1F). In contrast, tumors from the DCA group exhibited larger necrotic areas and fewer malignant cells, suggesting that inhibition of glycolytic metabolism suppresses tumor proliferation and promotes cell death (Fig. 1G–I). These results reinforce the hypothesis that glycolytic metabolism is crucial for tumor growth, and its inhibition can lead to increased tumor cell apoptosis. To further evaluate the impact of DCA and Rotenone on breast cancer liver metastasis, we utilized a 4T1-Luciferase cell line, which was injected into the livers of nude mice. The fluorescence intensity observed on the abdominal surface was notably stronger in the ROT-treated group, indicating that inhibition of aerobic respiration enhanced liver metastatic tumor growth (Fig. 1J–L; Supplementary Fig. 1C). In contrast, fluorescence was significantly reduced in the DCA-treated group comparing with the control group, suggesting that anaerobic respiration plays a larger role in supporting metastatic tumor expansion. Taking together, we recognized that anaerobic respiration metabolism contributes a powerful promotion on cancer cell survival.

### H3k18la and HDACs’ diversity in response to respiration metabolism

**Fig. 2:**
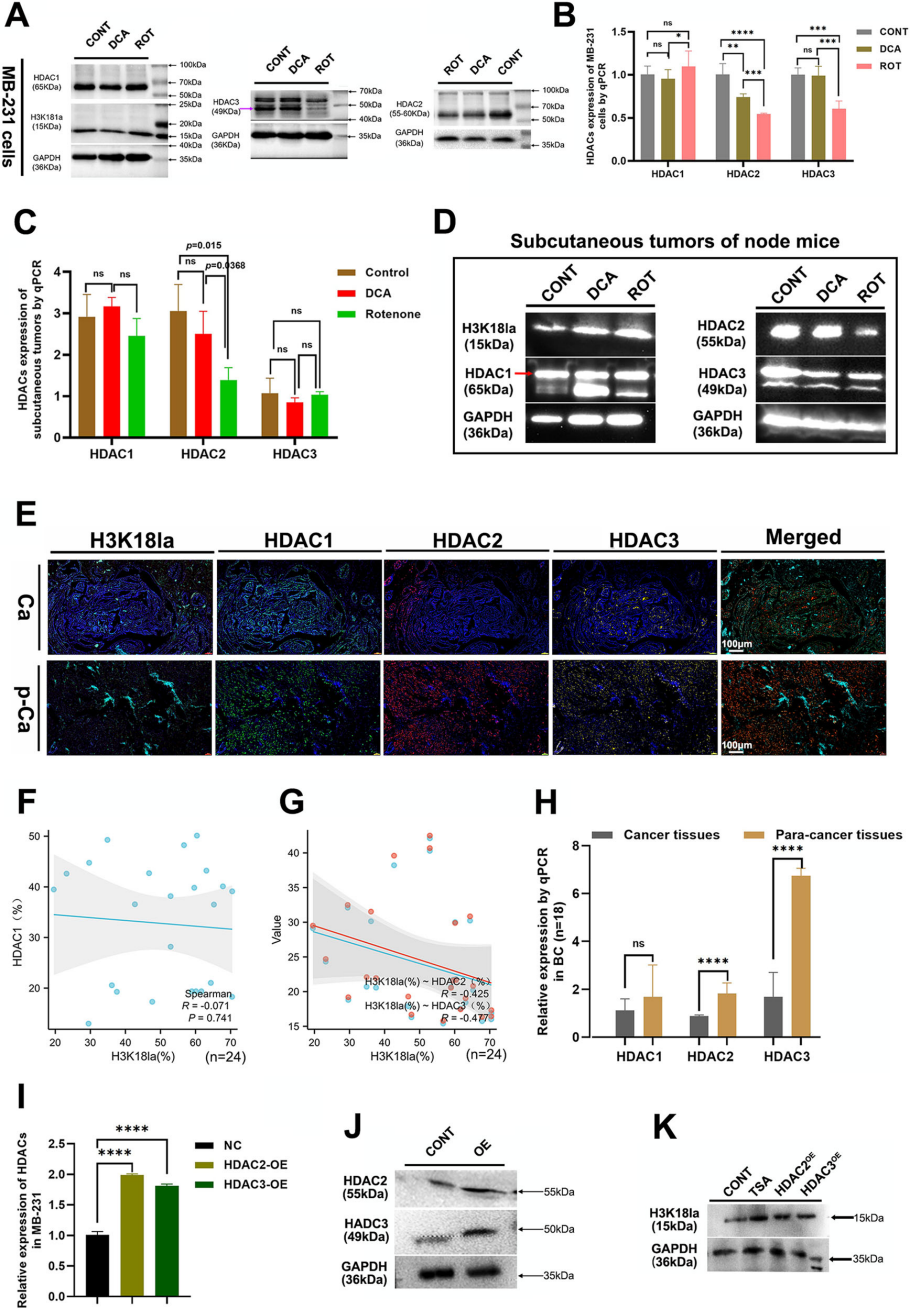
HDACs family regulated H3K18la expressions. **A** and **B** The expressions of H3K18la and HDACs family in MB-231 cells treated with DCA and Rotenone by qPCR and WB assays. **C** and **D** The expressions of H3K18la and HDACs family in subcutaneous tumors of nude mice treated with DCA and Rotenone by qPCR and WB assays. **E** The expressions of H3K18la and HDACs family in clinical breast cancer tissues by immunofluorescence staining (n-24). **F** and **G** Statistical Spearman’s analysis of correlations between H3K18la and HDACs family. **H** The expressions of HDACs family in clinical breast cancer tissues by qPCR assay (*n* = 18). **I** and **J** The expressions of HDAC2 and HDAC3 after transfecting overexpression plasmids into MB-231 cells. **K** The H3K18la expression in HDACs overexpression group, HDACs inhibitor group, and control group by WB assay. All experiments had three biology repetitions with similar results. Data are mean ± SD. *P < 0.05, **P ≤ 0.01, ***P < 0.001, ****P ≤ 0.0001.

Histone H3K18 lactylation (H3K18la) has emerged as a significant epigenetic modification driven by lactate, particularly during anaerobic respiration in cancer cells [21]. The histone deacetylase (HDAC) family plays a crucial role in regulating histone lactylation, an important post-translational modification associated with lactate metabolism [22]. To confirm their divergent expressions and function under anaerobic conditions of BC cells, we detected the H3k18la and HDACs (HDAC1/2/3) levels in MB-231 cells by qPCR and WB assays, showing that the level of H3K18la was significantly elevated in the ROT group compared to the DCA group and the control group (Fig. 2A, B). HDAC2 and HDAC3 levels cut down overtly in the ROT group compared with other groups, presenting that anaerobic metabolism inhibited HDAC2 and HDAC3 expressions. No significant differences in HDAC1 levels were observed across the experimental groups. Subsequently, in the subcutaneous tumor xenografts of nude mice, we examined the expression of H3K18la and HDACs following DCA and Rotenone treatment, indicating that H3K18la levels were significantly elevated in the ROT group compared to the control and DCA groups, but HDAC2 expression was lowest in the ROT group, while no statistically significant differences were observed for HDAC1 and HDAC3 across all treatment groups (Fig. 2C, D). These results suggested that H3K18la might have a negative correlation with HDAC2 at anaerobic respiration conditions in breast cancer cells. Next, expression of H3K18la and HDACs were measured by IF staining and qPCR assay in breast cancer tissues and para-cancerous tissues of patients (*n* = 24; Fig. 2E; Supplementary Fig. 1D), exhibiting that H3K18la expression was higher in BC tissues than that in BC para-cancerous tissues, while HDAC2 and HDAC3 decreased in BC para-cancerous tissues. The HDAC1 level still had no statistical significance which was coincident with outcomes in vitro and in vivo experiments. Linear Cox analysis revealed no significant correlation between HDAC1 and H3K18la expression levels. In contrast, both HDAC2 and HDAC3 showed a negative correlation with H3K18la expression (Fig. 2F, G). Meanwhile, the qPCR analysis of HDAC expression was also coincident with these by IF assay (Fig. 2H). To validate the regulatory role of HDAC2 and HDAC3 on H3K18la, we transfected MB-231 cells with HDAC2 and HDAC3 overexpression (OE) plasmids, alongside control and TSA-treated groups (Trichostatin A, an HDAC inhibitor) (Fig. 2I, J; Supplementary Fig. 1E). The results showed that H3K18la expression was significantly reduced in both HDAC2-OE and HDAC3-OE groups. Conversely, H3K18la levels were markedly increased in the TSA-treated group (Fig. 2K; Supplementary Fig. 1F). These results indicate that HDAC2 and HDAC3 are crucial regulators of histone H3 lactylation, actively reducing H3K18la levels by promoting delactylation processes.

### H3K18la-mediated PPARD expression in BC

**Fig. 3:**
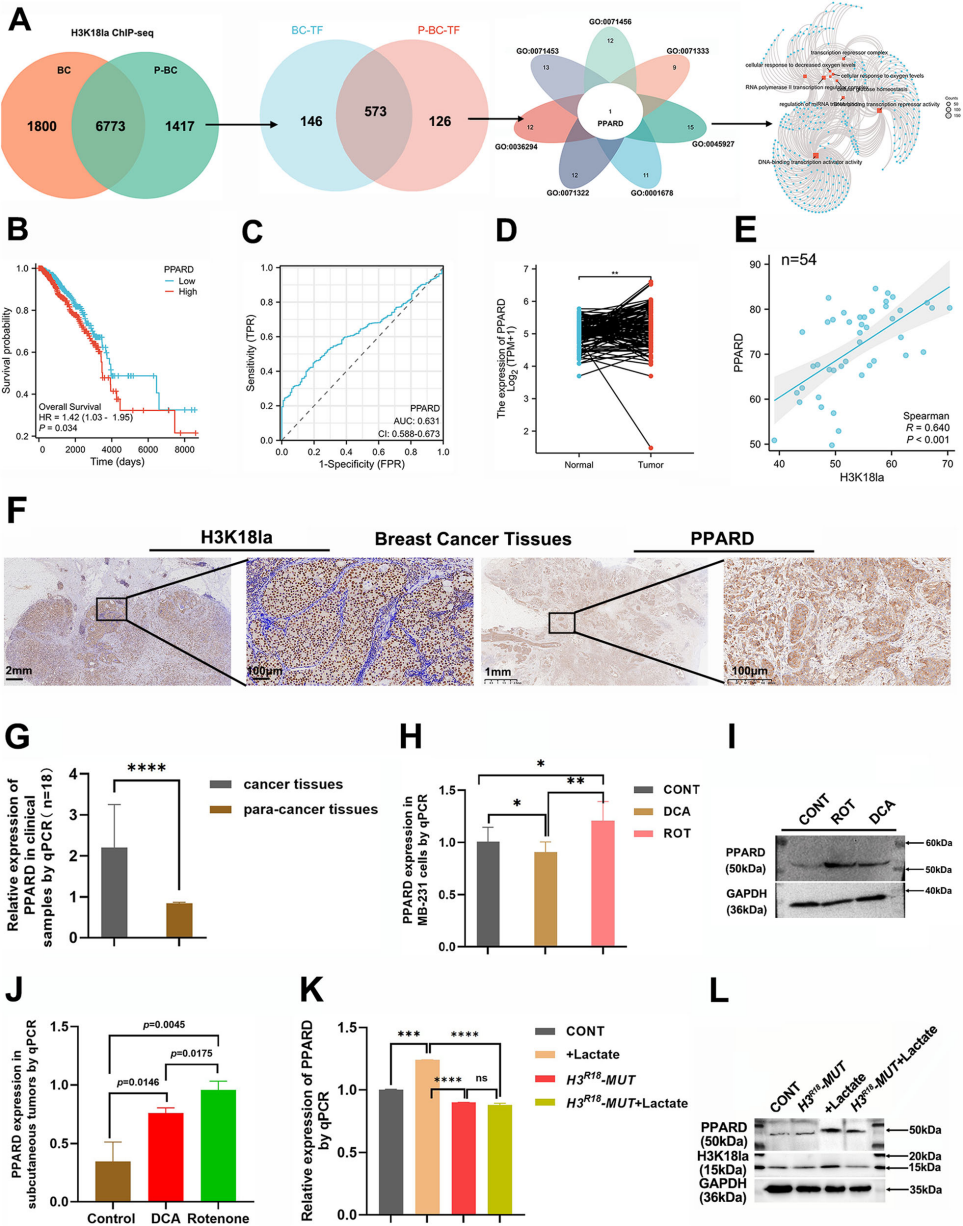
H3K18la promoted PPARD expression in breast cancer. **A** ChIP-seq with H3K18la antibody identified different genes in both BC and adjacent tissues, visualized using a Venn diagram. Transcription factors from these genes were analyzed using Homer 4.1, showing differences in a Venn diagram. PPARD, a transcription factor identified from KEGG data. **B**–**D** The overall survival curve, ROC curve, and expressions of PPARD in BC based on the TCGA database. **E** and **F** H3K18la and PPARD expressions in clinical breast cancer tissues with their correlative line by immunohistochemistry staining (*n* = 54). The scar bars were 2, 1, and 100 μm. **G** Relative expressions of PPARD in breast cancer and para-cancer tissues of clinical samples by the qPCR assay. **H**–**J** PPARD expressions in MB-231 cells and subcutaneous tumors of nude mice treated with DCA and Rotenone, respectively. **K** PPARD expressions were detected by qPCR and WB assays in H3^R18^ mutation group and plus lactate group. All experiments had three biology repetitions with similar results. Data are mean ± SD. *P < 0.05, **P ≤ 0.01, ***P < 0.001, ****P ≤ 0.0001.

To identify the downstream molecules regulated by H3K18la, we performed ChIP-seq analysis using an H3K18la antibody on BC and para-cancerous tissues. This analysis revealed 6773 shared genes, of which 573 were transcription factors. Venn clustering highlighted peroxisome proliferator-activated receptor delta (PPARD) as a key transcription factor associated with cellular responses to carbohydrate metabolism, hypoxia, and cell proliferation (Fig. 3A; Supplementary Tables 8, 9). Through TCGA database analysis, high expression of PPARD was found to correlate with poor prognosis in BC patients by which Kaplan–Meier survival analysis showed a significant association between elevated PPARD levels and reduced overall survival (*p* = 0.034; Fig. 3B). Additionally, the ROC curve indicated that PPARD has moderate predictive power, with an area under the curve (AUC) of 0.673 (Fig. 3C). Furthermore, PPARD was found to be highly expressed in tumor tissues compared to adjacent tissues based on the TCGA database (Fig. 3D). Then, we also detected the level of PPARD in clinical BC samples by IHC staining (*n* = 54; Fig. 3E, F) and qPCR assays (*n* = 18; Fig. 3G), suggesting that PPARD expression was higher in BC tissues, and was positively correlated with H3K18la expression via Spearman analysis (*p* < 0.001). Based on the clinical information analysis, the level of H3K18la was higher in over 45 years old than in young patients (*p* < 0.0001), and the more advanced the tumor stage, the higher H3K18la expression (Table 1). These findings elucidated that H3K18la was associated with poor prognosis in BC patients.

Moreover, the level of PPARD treated with DCA in MB-231 cells obviously decreased compared to that treated with Rotenone (Fig. 3H, I; Supplementary Fig. 1G). In subcutaneous tumor samples of nude mice, the PPARD tendency was coincident with that in MB-231 cells (*p* < 0.05; Fig. 3J). Based on these coherent results, we speculate that H3K18la may mediate the expression of PPARD. To verify this, we designed a mutant form of H3K18 plasmid, referred to as H3^R18^ (*H3*^R18^*-MUT*), and transfected it into 293T cells. Our findings demonstrated that the high expression of PPARD in the lactate group was diminished due to the interference from *H3*^R18^*-MUT*. Furthermore, the low level of PPARD observed in the *H3*^*R18*^*-MUT* group could not be rescued by adding lactate (Fig. 3K, L; Supplementary Fig. 1H).

### H3K18la/PPARD axis promoted cell proliferation via PI3K/AKT signaling pathway

**Fig. 4:**
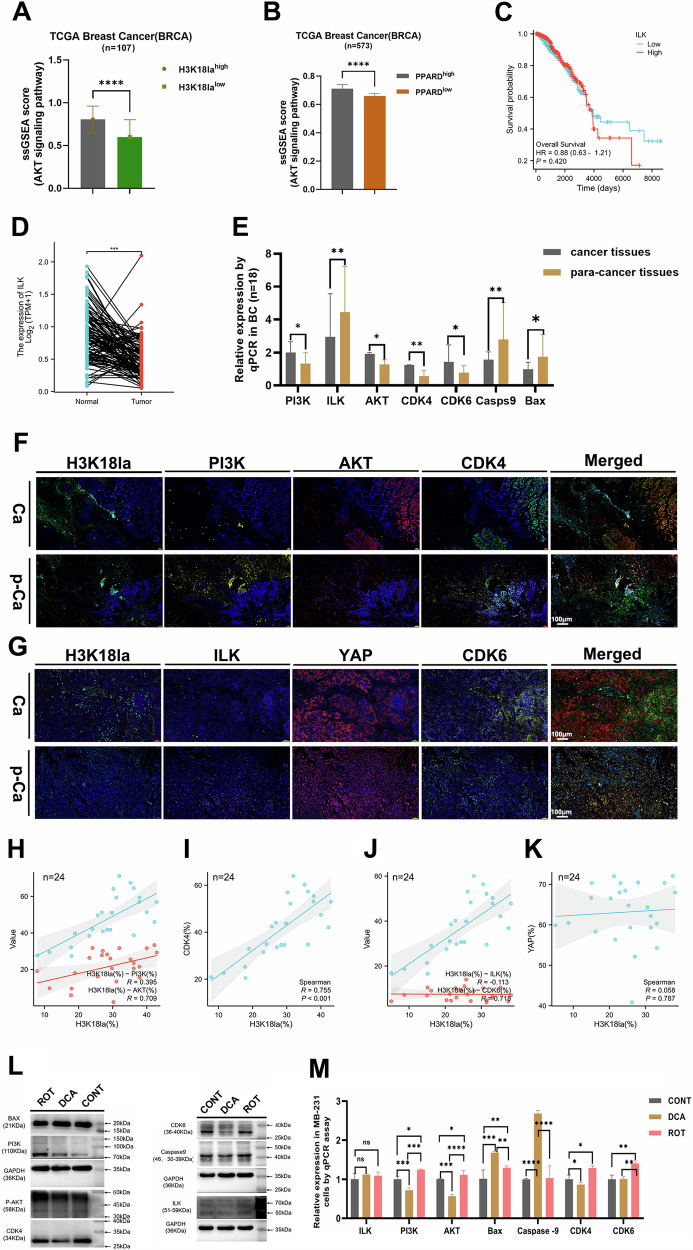
H3K18la/PPARD axis was associated with the PI3K/AKT signaling pathway. **A** and **B** High levels of H3K18la and PPARD in the TCGA-BRCA database were linked to the AKT signaling pathway based on ssGSEA scores. **C** and **D** The overall survival and expression of ILK in BC according to the TCGA database. **E**–**G** AKT signaling pathway relative markers in clinical breast cancer tissues and para-cancerous tissues by Qpcr (*n* = 18) and immunofluorescence staining (*n* = 24). **H**–**K** Spearman’s analysis of H3K18la and AKT signaling pathway relative markers based on the IF score. **L** and **M** PI3K/AKT signaling pathway relative markers expression in MB-231 cells with DCA and Rotenone treatments by qPCR and WB assays. All experiments had three biology repetitions with similar results. Data are mean ± SD. *P < 0.05, **P ≤ 0.01, ***P < 0.001, ****P ≤ 0.0001.

To explore the downstream signaling pathways regulated by the H3K18la/PPARD axis that contribute to breast cancer cell survival, we examined the KEGG database for components associated with the PPAR signaling pathway. Our analysis identified ILK and YAP as key downstream effectors of this pathway (Supplementary Fig. 1I). Importantly, ILK serves as a critical rate-limiting enzyme within the AKT signaling cascade, influencing key cellular processes such as survival, apoptosis, differentiation, and immune response regulation [23] (Supplementary Fig. 1J). Additionally, a thorough analysis of data from the TCGA database revealed a strong correlation between high expression levels of both H3K18la and PPARD and enhanced AKT pathway activity, as assessed by ssGSEA scores (Fig. 4A, B). These findings suggest a significant interplay between H3K18la and PPARD in modulating AKT signaling, which may facilitate BC progression. Next, we found that ILK expression was reduced in BC tissues and its high level was correlated with poor prognosis of BC patients (Fig. 4C, D). However, the PI3K/AKT signaling pathway was highly expressed in clinical BC samples by qPCR and IF staining assays (Fig. 4E–G; Supplementary Fig. 1K, L), and H3K18la was positively correlated with PI3K, AKT, CDK4 and CDK6 by Spearman’s analysis, respectively (Fig. 4H–J). The ILK level was negatively related to the H3K18la expression which was irrelevant to the YAP expression (Fig. 4K). Subsequently, to explore whether these aforementioned outcomes were consistent with in vitro (MB-231 cells) and in vivo experiments, we investigated expressions of AKT signaling pathway markers as well as cell survival markers, treated with DCA and Rotenone by qPCR and WB methods, showing that PI3K/AKT signaling pathway activated in the Rotenone treated which promoted cell proliferation and inhibited cell apoptosis (Fig. 4L, M; Supplementary Figs. 1M and 2A–D).

**Fig. 5:**
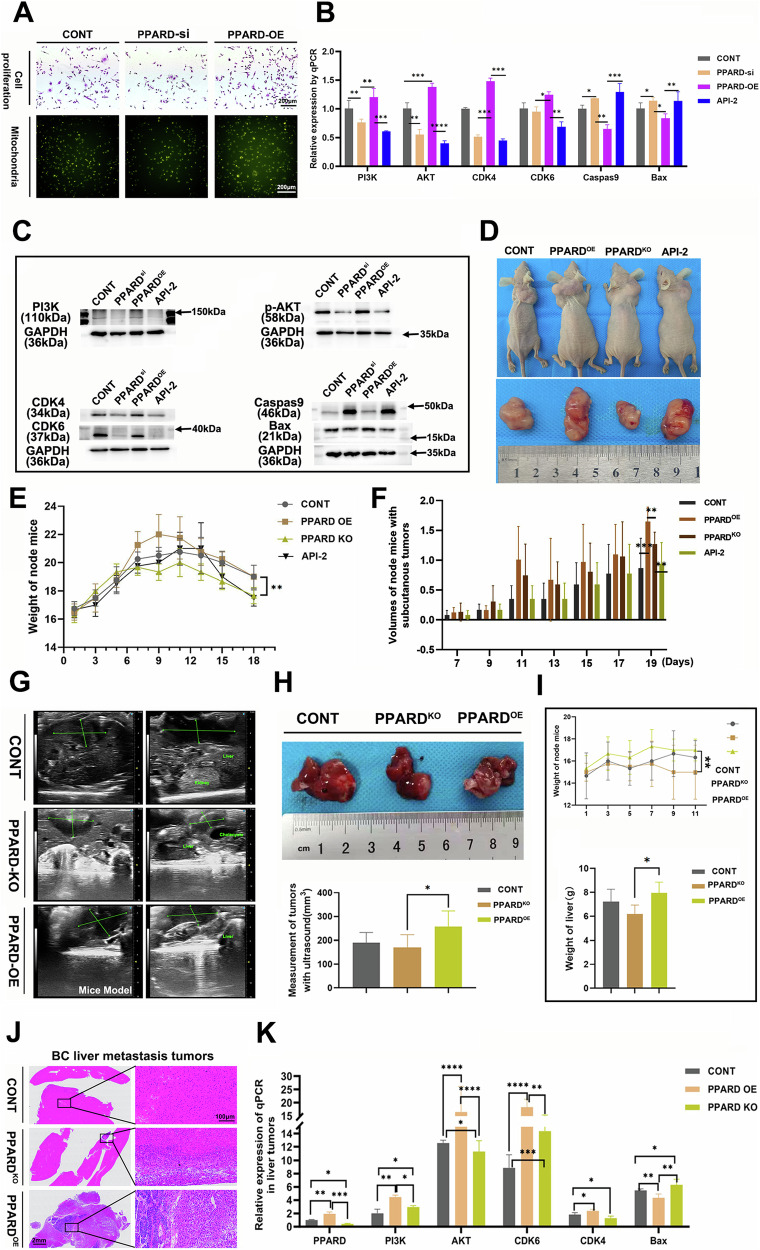
PPARD promoted BC proliferation via activating the PI3K/AKT signaling pathway. **A** Monoclonal proliferation assay and Mitochondria number of MB-231 cells after transfecting overexpression and silence PPARD plasmids. **B** and **C** The expressions of AKT signaling pathway markers in different groups by qPCR and WB assays. **D**–**F** The subcutaneous tumors of nude mice model with MCF-7 cells in different groups with weight and volume curves. **G**–**I** The breast cancer liver metastasis tumors with MCF-7 cells by ultrasound measurements in different groups with weight and volume curves. **J** Hematoxylin and Eosin staining showed differences in liver tumor intensity. **K** The expressions of AKT signaling pathway markers in liver metastasis tumors by qPCR assay. All experiments had three biology repetitions with similar results. Data are mean ± SD. *P < 0.05, **P ≤ 0.01, ***P < 0.001, ****P ≤ 0.0001.

To elucidate the role of PPARD in the regulation of the AKT signaling pathway, we constructed PPARD overexpression (PPARD-OE) plasmids and silence RNA (PPARD-si) plasmids to transfect MB-231 cells (Supplementary Fig. 2E, F), suggesting that PPARD-OE could accelerate the proliferation and mitochondria of cancer cells compared to the PPARD-si group (Fig. 5, A; Supplementary Fig. 2G, H). In addition, we performed WB and qPCR analyses across four experimental groups: control, PPARD overexpression (PPARD-OE), PPARD knockdown (PPARD-si), and API-2 (an AKT pathway inhibitor). Our analysis of key biomarkers involved in the PI3K/AKT signaling pathway, including PI3K, AKT, CDK4, and CDK6, revealed that the expression levels of proliferative markers were significantly elevated in the PPARD-OE group compared to both the PPARD-si group and the API-2 group (*p* < 0.05) while apoptotic markers—Bax, and Caspase 9 decreased (Fig. 5B, C; Supplementary Fig. 2I). Specifically, the upregulation of AKT-related markers in the PPARD-OE group underscores the potential role of PPARD in promoting AKT signaling activation. In vivo models, the subcutaneous tumor of the PPARD-OE group was bigger than that in the PPARD knock-out group and API-2 group compared to the control group (Fig. 5D–F), as well as that the liver metastatic tumor in the PPARD-OE group was bigger than the other groups with MCF-7 cells (Fig. 5G–J). The PI3K, AKT as well as the proliferative markers-CDK4 and CDK6 expression, increased in the PPARD-OE group while the Bax level decreased simultaneously (Fig. 5K). Thus, these findings provide compelling evidence that PPARD enhances the proliferation of cancer cells via activating the PI3K/AKT signaling pathway.

### The mechanism of PPARD in active transcription

**Fig. 6:**
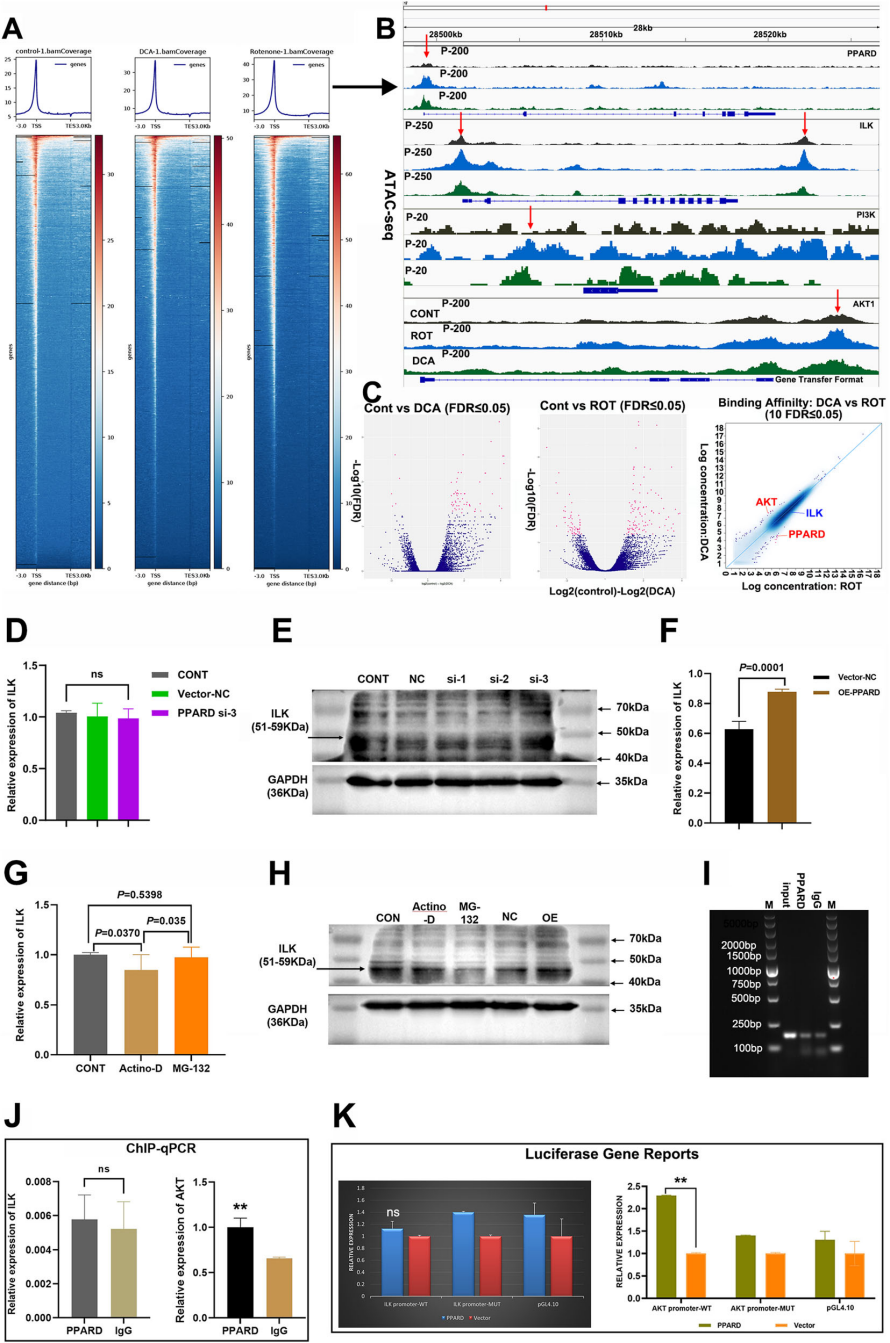
The mechanism of PPARD interacted with ILK and AKT in breast cancer. **A** and **B** Heatmaps of gene coverage in subcutaneous tumors in control, DCA, and ROT groups, and IGV software were used to visualize the gene accessibility. **C** Heatmaps comparing gene expressions in DCA and ROT groups compared to the control group. **D**–**H** The expression of ILK in MB-231 cells with overexpression and silence of PPARD or treated with Actinomycin D and MG-132 by qPCR and WB assays. **I** and **J** Successful qPCR electrophoresis results of ChIP assays for PPARD. ILK and AKT expressions were detected with PPARD antibody compared to IgG control. **K** Luciferase gene reports showed significant luciferase activity in the AKT promoter-wt group, with no difference in other groups after co-transfecting with wild-type promoter sequences and mutations in 293T cells. Each ILK plasmid group showed no differences. Data are mean ± SD. *P < 0.05, **P ≤ 0.01, ***P < 0.001, ****P ≤ 0.0001.

Thereby, to highlight the need for further investigations to unravel the specific molecular mechanisms through which PPARD exerts its effects on AKT signaling and its broader implications in cancer biology, we utilized distinct samples of subcutaneous tumors of nude mice after treated with DCA and Rotenone for ATAC-seq testing to study chromatin accessibility and the regulatory landscape of the genome. The heatmap analysis of ATAC-seq data revealed that the Rotenone-treated tumor group exhibited significantly greater chromatin openness compared to both the DCA-treated group and the control tumors (Fig. 6A). This increased accessibility correlates with heightened transcriptional activity of involved genes, suggesting that the predominance of anaerobic respiration in tumors treated with Rotenone may create a more favorable chromatin environment for gene activation. The ATAC-seq data were visualized using the integrative genomics viewer (IGV) software, with the results indicating that the Rotenone-treated experimental group exhibited higher accessibility of genomic regions associated with the genes PPARD, ILK, PI3K, and AKT compared to the other two experimental groups (Fig. 6B). This enhanced chromatin accessibility in the ROT group suggested a potential increase in the transcriptional regulation of PI3K/AKT signaling pathways. Furthermore, the Volcano plot directly performed DEGs between the DCA group and control group, the ROT group and control group, DCA group and ROT group, indicating that PPARD and AKT levels increased while ILK level showed no significant differences (Fig. 6C). To analyze the interactions among PPARD, ILK, and AKT at the genetic level, the expression of ILK was detected with PPARD-OE and PPARD-si interference MB-231 cells by qPCR and WB assays, suggesting that the high level of ILK performed in the PPARD-OE group compared to the PPARD-si group and vector-NC group (*p* = 0.0001; Fig. 6D, E). Subsequently, treatment of MB-231 cells with Actinomycin D resulted in a significant downregulation of ILK mRNA levels compared to the control group, indicating that transcriptional regulation of the ILK gene is affected by this agent. In contrast, no significant differences in ILK mRNA expression were observed in the MG-132 treatment group when compared to the control (Fig. 6F, G). Additionally, WB analysis revealed that ILK protein levels were markedly increased in the PPARD-OE group. Conversely, ILK protein expression was significantly reduced in the MG-132-treated cells, suggesting that proteasomal inhibition may impact ILK protein stability and expression, and PPARD might influence the ILK at the DNA transcription level (Fig. 6H). Based on the analysis of the aforementioned results, we first performed a search for the motif sequence of PPARD as a transcription factor. Utilizing predictions from the JASPAR database (Supplementary Fig. 3A, B), we identified structural domain sequences that suggest potential binding sites for PPARD on the ILK and AKT genes. Subsequently, we conducted ChIP assays combined with qPCR to assess the expression levels of AKT and ILK in the context of PPARD activation (Fig. 6I). The results demonstrated that AKT expression was significantly upregulated in the PPARD experimental group, while ILK expression remained unchanged (Fig. 6J). Furthermore, we constructed a luciferase reporter assay to evaluate the transcriptional activity of the ILK and AKT promoter revealing that the fluorescence intensity of the AKT promoter-wild type (WT) group was markedly higher than that of both the AKT promoter-mutant (MUT) group and the pGL4.10 control group, while there was no statistical significance of ILK promoter-WT and MUT groups. This finding confirms that PPARD interacts with the AKT promoter and facilitates its transcription (Fig. 6K). In summary, our results suggest that PPARD positively regulates AKT expression by directly binding to its promoter, while ILK expression does not appear to be affected by PPARD activation. These data provide new insights into the regulatory mechanisms by which PPARD may influence key signaling pathways involved in cancer progression. Further investigations are warranted to explore the functional implications of this interaction in greater depth.

### Protein phosphorylation omics for AKT analysis

**Fig. 7:**
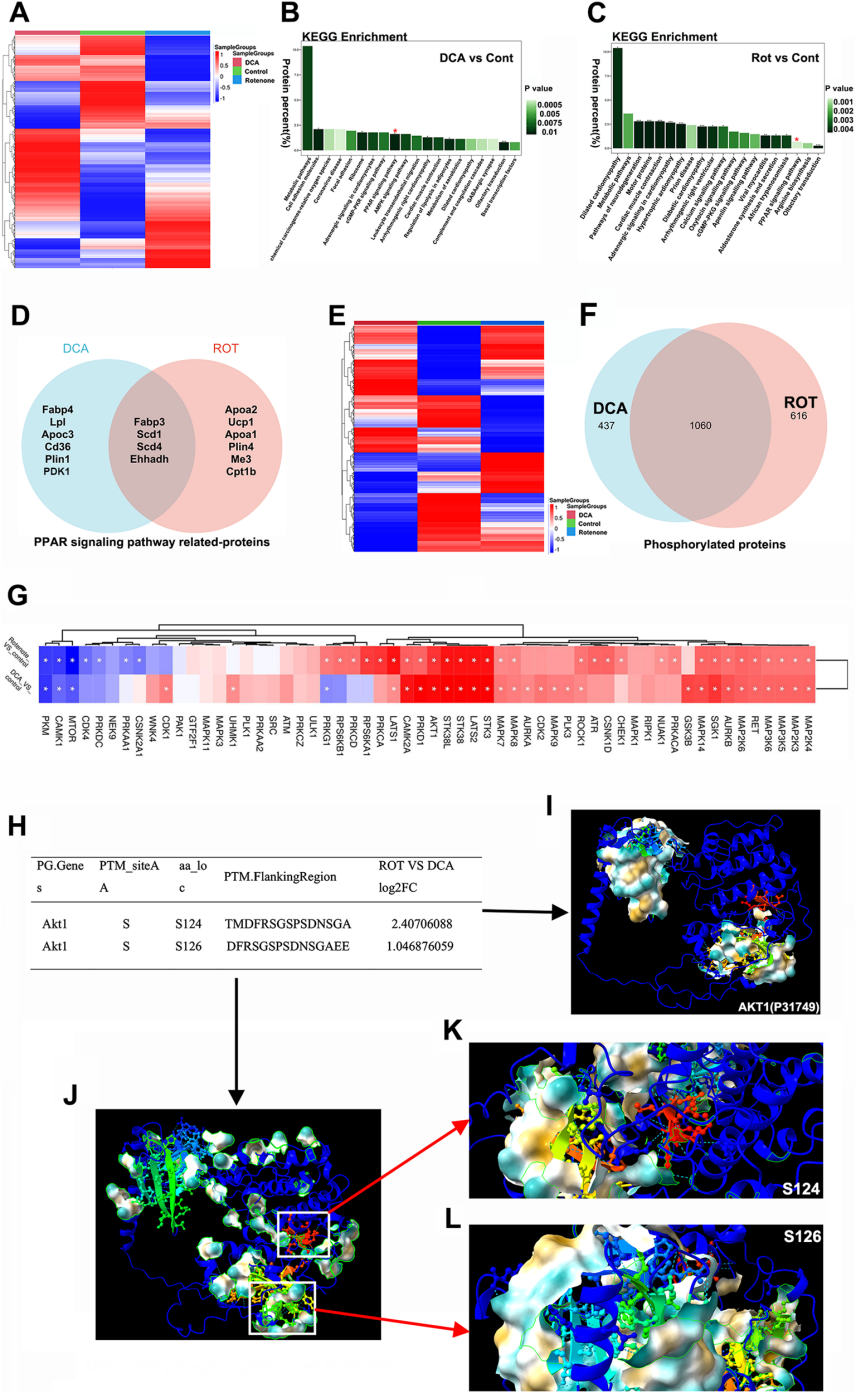
Proteomics and phosphorylated modification analysis in glycolysis metabolism. **A** Heatmap showing proteomic differences among DCA, Rotenone, and control groups in subcutaneous tumors. **B** and **C** KEGG clustering analysis of different proteins in DCA, Rotenone, and control groups. **D** Venn diagram clustering proteins from DCA and ROT groups related to the PPAR signaling pathway. **E** Heatmap showing differences in phosphorylated proteomics among DCA, Rotenone, and control groups in subcutaneous tumors. **F** Venn diagram clustering proteins from DCA and ROT groups. **G** Top 60 different proteins in DCA and ROT groups. **H** Two AKT1 serine phosphorylation sites were detected in the ROT group compared to the DCA group. **I**–**L** Three-dimensional structures of serine 124/126 phosphorylation sites displayed using ChimeraX software.

To systematically investigate the molecular alterations associated with the reprogramming of energy metabolism in cancer cells exhibiting abnormal respiration, we conducted quantitative proteomics on subcutaneous tumors derived from breast cancer models. Our analysis identified 751 distinct proteins in the ROT group compared to the control group, and 618 proteins in the DCA-treated group relative to the control (Table 2). The heatmap analysis clearly delineated differences among the three groups, corresponding to varying respiratory conditions of the cancer cells (Fig. 7A). To comprehend the functional roles of these proteins, we performed a comprehensive bioinformatics analysis utilizing the KEGG database, which revealed significant involvement of the PPAR signaling pathway in both the DCA and ROT groups (Fig. 7B, C). A volcano plot disclosed that in the DCA group, 47 proteins were downregulated and 45 were upregulated, with a Log Fold Change (Log FC) greater than ± 3. In the ROT group, 41 proteins were upregulated while 67 were downregulated (Supplementary Fig. 3C, D). Notably, under anaerobic conditions, proteins such as fatty acid binding protein (FABP), Lopadostoma linospermum (Lpl), perilipin 1 (Plin1), pyruvate dehydrogenase kinase 1 (PDK1), and CD36 were regulated by PPARD, while malic enzyme 3 (Me3), uncoupling protein 1 (Ucp1), perilipin 4 (Plin4), and carnitine palmitoyltransferase 1B (Cpt1b) were influenced by PPARA (Fig. 7D). Following this, we investigated the quantitative phosphoproteomics to identify functionally phosphorylated proteins involved in glycolysis (Fig. 7E). Venn diagram analysis illustrated the presence of 437 specific phosphorylated proteins in the DCA group and 616 in the ROT group (Fig. 7F). Among these, the top 60 proteins exhibiting significant *p*-values revealed high expression levels of phosphorylated AKT1, along with members of the STK family and LATS2 under the altered respiratory metabolism of cancer cells (Fig. 7G). Additionally, KEGG pathway analysis of the differentially phosphorylated proteins indicated a prominent role in the PPAR signaling pathway (Supplementary Fig. 3E, F). ChimeraX software is used to visualize AKT1 structures in three dimensions, revealing significant phosphorylation at serine-124 (S124) and serine-126 (S126) (Fig. 7H–L). Moreover, we predicted potential protein motif sequences surrounding the modification sites, which included 15 amino acids flanking both the most upregulated and downregulated regions in the DCA versus control and Rotenone versus control comparisons (Supplementary Fig. 3G, H).

## Discussion

Histone lactylation, a post-translational modification involving the addition of lactate groups to lysine residues on histones [5, 24]. This modification is catalyzed by specific enzymes. Histone lactylation plays a critical role in regulating tumorigenesis, immune response, and energy metabolism within the tumor microenvironment [4, 5, 25, 26]. The modification affects gene expression by altering chromatin structure, thereby linking metabolic changes to epigenetic regulation. In an anaerobic metabolic environment, tumor cells exhibit distinct changes in chromatin accessibility that can enhance the transcription of various genes. The low oxygen conditions typical of many tumor microenvironments lead to adaptations in cellular metabolism, allowing cancer cells to thrive despite limited oxygen availability. These adaptations often involve a shift toward anaerobic respiration, which not only alters energy production pathways but also influences chromatin structure and gene expression. According to our study, we confirmed that glycolysis metabolism in BC cells accelerated the level of H3K18la and abrogated the expressions of HDAC2 and HDAC3 which subsequently might be evaluated as the “eraser” of histone-3 lactylation. Consistent with Fan et al.’s conclusions, HDAC2 could construct a feedback loop with H3K9 lactylation in endothelial cells by regulating VEGF-induced angiogenesis [9, 27]. The HDAC family of proteins, known for their role in histone deacetylation and chromatin remodeling, may potentially interact with histone lactylation to regulate gene expression and cellular processes [28]. Understanding the crosstalk between histone lactylation and HDACs could provide insights into novel mechanisms of epigenetic regulation and their implications for health and disease. Furthermore, the comprehensive molecular mechanism of H3K18la is proven to enhance the openness of chromatin and expose extensive promoter regions of functional genes, especially under the abnormal respiration conditions of cancer cells; this phenomenon is presented eminently. The rapid response of glycolysis in cancer cells rescues them to survive prolonged respiratory metabolic abnormalities. This hypothesis could be delineated from the outcomes of cell proliferation and apoptosis by the flow cytometry assay in our study. Melkonian and Schury discussed the importance of anaerobic glycolysis in hypoxic tumor environments, where it rapidly produces ATP and lactate, supporting cancer cell survival and angiogenesis [29]. In contrast, You et al. studied a protein from *Hemerocallis citrina* Borani that inhibited hepatocellular carcinoma by triggering apoptosis and regulating glycolysis, disrupting cancer cell energy production and survival [30]. Additionally, Barba et al. reviewed strategies to target the Warburg effect in cancer therapy, emphasizing the disruption of glycolysis pathways to reduce tumor growth and enhance treatment efficacy [31]. Together, these studies highlight various metabolic pathways and their implications for cancer progression and treatment, but they have contradictory results which give us more nonredundant and ulterior explorations.

Peroxisome proliferator-activated receptor delta (PPARD) plays a significant role in various disease mechanisms through its regulation of lipid metabolism, inflammation, and cell proliferation [11, 13–15]. It enhances the Wnt/β-catenin pathway, increasing β-catenin levels to stimulate target genes involved in cell growth [17]. Overexpression of PPARD in colorectal cancer leads to enhanced tumor growth and survival [32]. Inhibiting PPARD can disrupt these pathways, reduce tumor progression, and improve chemotherapy efficacy. Similarly, in the PI3K-AKT pathway, the exact intermediates and feedback mechanisms that PPARD might engage with remain to be clarified. In terms of our study, we found a significant transcriptional mechanism of PPARD in regulating the PI3K/AKT signaling pathway by which PPARD had combined motif sequences with both ILK and AKT in promoter regions of DNA, but only AKT could be promoted. This encouraging result changes our conceptions of the PPAR signaling pathway in the KEGG database, showing that both ILK and AKT are downstream factors that impact cell survival. Remarkably, the referenced study demonstrates that PPARD activation can attenuate Angiotensin II (Ang II)-induced senescence in vascular smooth muscle cells (VSMCs) by up-regulating PTEN, which suppresses the PI3K/AKT pathway and reduces reactive oxygen species generation [16]. In VSMCs under oxidative stress from Ang II, PPARD’s up-regulation of PTEN acts as a protective mechanism by inhibiting AKT signaling [16]. In contrast, in cancer cells, PPARD may enhance AKT signaling to support proliferative and survival pathways, potentially due to different regulatory mechanisms or cofactors. Additionally, ligand-activated PPARD might exhibit different effects compared to overexpression without specific ligands, and differential gene expression and pathway interactions in various cell types further explain the variability in PPARD’s effects. Hitherto, the regulation of PPARD itself—how it is activated or repressed by upstream signals—is not completely mapped. Identifying the external signals, such as hormones, nutrients, or stress conditions, and the intracellular mediators that influence PPARD activity could provide deeper insights into its role in cancer and other diseases.

Proteomics identifies biomarkers for cancer diagnosis, prognosis, and treatment by analyzing protein expression, enabling early detection and personalized therapy [33–37]. To deeply understand the changes of proteins under the abnormal respiration conditions, we detected the proteomics and phosphorylate modifications with DCA and ROT group tumors, verifying that PPAR signaling pathway-related proteins had obvious differences and phosphorylated AKT1 contributed to activate PI3K/AKT signaling pathway in glycolysis metabolism of BC tumors. Together, it suggests that glycolysis metabolism has power to sustain breast cancer cells survival via activating the AKT phosphorylation process.

**Fig. 8:**
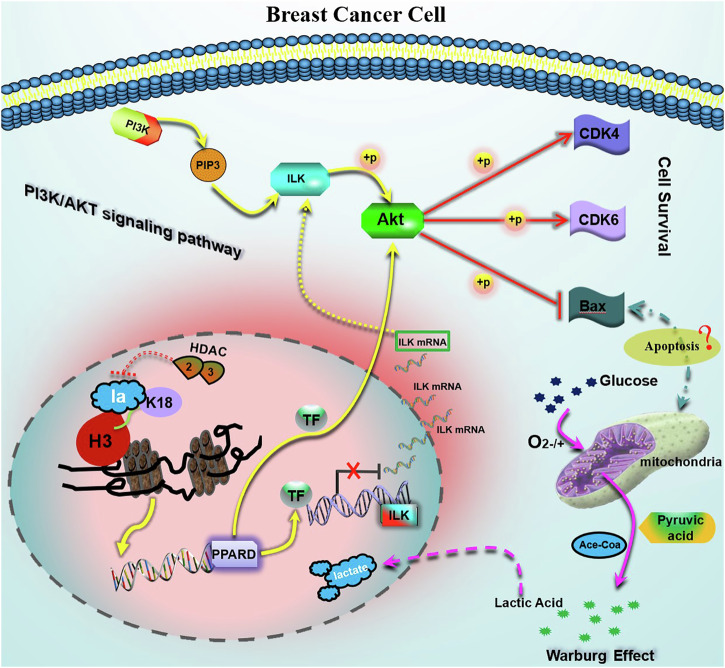
The mechanism simulation diagram of H3K18la mediating the PPARD/AKT signaling pathway.

Despite the significant insights gained, our study still has several limitations that need to be addressed in future research. The relatively small sample size limits the generalizability of our findings, necessitating validation with larger cohorts. Additionally, while our study indicates the therapeutic potential of targeting the H3K18la/PPARD/AKT axis, in vivo studies using animal models treated with specific inhibitors are necessary to assess the efficacy and safety of such treatments. Future research should focus on expanding sample sizes, conducting comprehensive functional studies, investigating detailed molecular mechanisms, and exploring the therapeutic potential of targeting the H3K18la/PPARD/AKT axis in preclinical models (Fig. 8).

## Conclusion

Histone lactylation is a significant epigenetic modification in breast cancer (BC), impacting key pathways that promote tumor survival and growth. ChIP-seq and bioinformatic analyses revealed that H3K18la regulates 6773 genes, with PPARD identified as a key transcription factor involved in responses to carbohydrates, hypoxia, and cell growth. Our research indicates that H3K18la boosts PPARD expression, which subsequently activates the PI3K/AKT signaling pathway, maintaining cell survival and proliferation in BC. The inverse relationship between H3K18la and HDAC2/3 levels shows that HDACs erase histone lactylation. Suppressing HDAC2 and HDAC3 raises H3K18la levels, maintaining the PPARD/AKT signaling axis. Furthermore, glycolytic metabolism via the “Warburg effect” aids cancer cell survival in hypoxia by increasing PPARD and activating the AKT pathway. In vivo and in vitro experiments show that inhibiting glycolysis with DCA reduces tumor growth and induces apoptosis, further confirming the role of the H3K18la/PPARD/AKT axis in BC. The link between PPARD expression and chromatin accessibility underscores the transcriptional regulation mechanism, with PPARD specifically increasing AKT transcription but not ILK. In addition, AKT1 phosphorylation at serine-124 and serine-126 may contribute a critical role in activating the AKT signaling pathway.

## Material and methods

### Clinical samples

Fresh BC and para-cancerous tissues were obtained from patients who underwent surgery at the breast and thyroid department of Shandong Provincial Third Hospital from September 2023 to June 2024 (54 samples). Two expert pathologists confirmed all diagnoses according to the American Joint Committee on Cancer guidelines. The paraffin sections of BC and para-cancerous tissues were obtained from the pathology department between January 2021 and June 2023 (123 paired samples). The study was approved by the Ethics Committee of Shandong provincial third hospital (KYLL-2023057), and all consents were gained from patients.

### Cell culture

MDA-MB-231 (MB231, ZQ0118, Zhongqiaoxinzhou cell company, Shanghai, China), 4T1 (ZQ0201), and 4T1-Luciferase (LZQ0016) cell-lines were purchased and cultured in Dulbecco’s modified Eagle medium supplemented with 10% fetal bovine serum (Biological Industries, Kibbutz Beit HaEmek, Israel) in the presence of penicillin, streptomycin and l-glutamin (Corning, New York, NY, USA) at 37 °C, 5% CO_2_ humidified condition while 293T cell-line (ZQ0033) was cultured in complete medium (ZM0033) in the laboratory of Shandong provincial third hospital. MB-231 cell-line was used for cellular experiments with 2.5 μm dichloroacetic acid (DCA) and 5 μm Rotenone (ROT), as well as the dimethyl sulfoxide (DMSO) controlling. 293T cell-line was utilized for plasmid transfection experiments, and the Luciferase gene reports experiment. Human PPARD knockout MCF7 cell-line (ab289456) was purchased from Abcam company of Shanghai Province and used to establish a subcutaneously implanted tumor model and liver metastatic tumor model of nude mice. These cell-lines were tested for mycoplasma contamination and characterized by short tandem repeat and Q-band assays.

### Real-time quantitative reverse transcription PCR

Total RNA was extracted from cultured cells using the Easy Pure RNA Isolation Kit (R0018S; Beyo Blu biology company, Shanghai, China) and reverse transcribed RT Kit (D7153; BeyoRT M-MuLV). The cDNA was preceded by real-time PCR with gene-specific primers in the presence of qPCR Master Mix (BeyoRT II cDNA). PCR reactions were performed in triplicate, and the relative amount of cDNA was calculated by the comparative CT values using the β-actin as an internal reference (Supplementary Tables 1, 2).

### Chromatin immunoprecipitation (ChIP)-PCR assay

MB-231 cells were crosslinked with 4% paraformaldehyde and sonicated (JY96-IIN, Xinzhi company, Ningbo, China). Chromatin was immunoprecipitated with antibodies against PPARD (ab178866) and control IgG (ab172730). Antibody–chromatin complexes were pulled down with Protein A + G Agarose/Salmon Sperm DNA, washed, and then eluted. After crosslink reversal and proteinase K treatment, immunoprecipitated DNA was extracted with phenol–chloroform, and ethanol precipitated [38]. The DNA fragments were further analyzed by qPCR of ILK and AKT.

### Immunohistochemistry (IHC) and immunofluorescence (IF) assays

Paraffine samples of BC and para-cancerous tissues, as well as animal tumor tissues, were selected to retrieve antigens, blocking non-specific binding, and incubating with a primary antibody (H3K18la, PTM-1406RM, 1:400 dilution, JINGJIE biology company, Hangzhou, China; PPARD, ab178866, 1:400) specific to the target antigen. A secondary antibody (IgG H&L, ab150077, 1:1000 dilution), usually enzyme-conjugated or fluorescently labeled, is then applied. Detection is achieved through colorimetric or fluorescence methods, followed by counterstaining with DAPI (Beyotime, China) for cell nuclei. The stained slides are examined under a microscope (3DHISTECH, PANNORAMIC DESK/MIDI/250/1000, Hungary) to analyze the presence and localization of the positively staining cells. CaseViewer software was used to browse the whole figures and screen to be stored (Supplementary Table 3).

### Western Blot assay

Proteins are extracted from cells or tissues and quantified (RIPA lysis buffer, HR0282, BIOLAB, Beijing, China), and then separated by size using sodium dodecyl sulfate–polyacrylamide gel electrophoresis (SDS–PAGE). Following separation, the proteins are transferred to a membrane (PVDF), and is then blocked with blocking buffer (YT067, BIOLAB, Beijing, China). Next, the membrane is incubated with a primary antibody specific to the target protein (Supplementary Table 4). After washing off, incubated with a secondary antibody (HRP Goar Anti-Rabbit, AS014, ABclonal, 1:5000; Anti-Mouse, AS003, 1:5000). The target protein is then visualized by adding a BalbECL Plus (YT060, BIOLAB, Beijing, China), usually a chemiluminescent output (FluorChem R, Purnoson, USA), which is then captured and analyzed by Image J software.

### Hematoxylin and Eosin (H&E) staining

Deparaffinization and rehydration of paraffin samples are performed by treating with xylene and alcohol series. Next, the tissue is stained with hematoxylin and eosin to color the cytoplasm and extracellular matrix pink. This is followed by dehydration through increasing alcohol concentrations, clearing in xylene, and finally mounting the tissue with a coverslip for microscopic examination (KF-PRO-005-EX, KFBIO, Ningbo, China).

### Chromatin immunoprecipitation sequence (ChIP-seq)

ChIP experiment and high through-put sequencing and data analysis were conducted by Seqhealth Technology Co., LTD (Wuhan, China). Fresh BC and para-cancerous tissues were fixed in 4% formaldehyde, and 0.125 M glycine was added to stop the crosslinking reaction. After collecting the nucleus and sonicating to fragment chromatin DNA, immunoprecipitation was performed with an H3K18la antibody (IP group), and a 10% sample was incubated with rabbit IgG (Cell Signaling Technology) as a negative control. DNA libraries were prepared by using the VAHTS Universal DNA Library Prep Kit for Illumina V3 (Catalog No. ND607, Vazyme). The library was enriched for 200–500 bps fragments, quantified, and sequenced on DNBSEQ-T7 sequencer (MGI Tech Co., Ltd. China) with PE150 model [39]. Raw sequencing data was first filtered by Trimmomatic, and They were mapped to the human reference genome. Bedtools was used for peak annotation and peak distribution analysis. The differentially binding peaks were identified by a python script with Fisher’s test. Motif analysis was conducted with Homer 4.1.

### Bioinformatics analysis

Gene ontology (GO) analysis and Kyoto Encyclopedia of Genes and Genomes (KEGG) enrichment analysis for annotated genes were both implemented by KOBAS software 2.1.1 with a corrected *P*-value cutoff of 0.05 to judge statistically significant enrichment. Visualizations, including different gene heatmaps, ROC curves, overall survival curves, and Cox regression analysis, were accessed using the Xiantao database (https://www.xiantaozi.com) based on the Cancer Genome Atlas (TCGA) database.

### Flow cytometry assay

Cells were harvested, washed with PBS, and then fixed with 70% ethanol at 4 °C for 30 min. After fixation, cells were washed again with PBS and resuspended in a staining solution containing propidium iodide (PI) and RNase A. For apoptosis detection, Annexin V-FITC and PI double staining was performed according to the manufacturer’s instructions. Samples were incubated in the dark at room temperature for 30 min. Flow cytometric analysis was conducted using a flow cytometer equipped (BD FACS Celesta, USA), and data were analyzed with FlowJo software to determine the proportion of proliferating and apoptotic cells.

### Cell monoclonal proliferation assay

Single cells were isolated by limiting dilution and seeded into six-well plates, with one cell per well. The cells were cultured in a complete growth medium and incubated at 37 °C in a humidified atmosphere with 5% CO_2_. Over a period of 10–14 days, cell colonies were monitored and assessed for proliferation. Colonies were fixed with 4% paraformaldehyde for 20 min at room temperature and then stained with 0.5% crystal violet solution for 30 min to visualize the colonies.

### Transfection

Cells were seeded into a six-well plate at a density of 2 × 10^5^ cells per well and incubated overnight to reach 90% confluence. For each well, plasmid DNA (2 μg PPARD-OE; 2 μg PPARD-si; 2.5 μg H3K18-mut/wt; 4 μg ILK/AKT-mut/wt) was diluted in 250 µL of serum-free medium and mixed gently. Separately, 10 µL of transfection reagent (e.g., Lipofectamine 2000) was diluted in 250 µL of serum-free medium and incubated for 5 min at room temperature. The diluted DNA and transfection reagent were then combined and incubated for 20 min to form DNA-transfection reagent complexes. The complexes were added dropwise to the cells, which were then incubated at 37 °C in a CO_2_ incubator. After 6 h, the medium was replaced with a fresh complete growth medium. Transfection efficiency was assessed 24–48 h post-transfection by observing the expression of a reporter gene (e.g., GFP) under a fluorescence microscope (Axio Imager.M2, ZEISS, USA) (Supplementary Fig. 1A, B).

### Transposase-accessible chromatin with high throughput sequencing (ATAC-seq)

Nuclei are isolated from nude mice subcutaneous tumors. The transposase enzyme Tn5 is then added, which inserts sequencing adapters into accessible regions of the chromatin. Following transposition, the DNA is purified and subjected to PCR amplification (MolPure® PCR Purification Kit PCR) to enrich for adapter-ligated fragments (Supplementary Table 5). The amplified DNA is then purified again and quantified (TruePrep DNA Library Prep Kit V2 for Illumina, TD501). Next, the library is sequenced using high-throughput sequencing platforms. Finally, the sequencing data is analyzed to identify open chromatin regions, providing insights into chromatin accessibility and regulatory element locations (VAHTS DNA Clean Beads, N411) [40]. This detection was constructed by the IGENECODE company in Beijing, China.

### Luciferase gene reports

293T cells are transfected with a plasmid containing a luciferase reporter gene under the control of a promoter of interest. After allowing time for expression, the cells are lysed to release the luciferase enzyme (Thermo Scientific). Next, a luciferase substrate is added to the cell lysate. The luciferase enzyme catalyzes a reaction with the substrate, producing light. The emitted light is then measured using a luminometer. The intensity of the light is proportional to the activity of the promoter, providing insights into gene expression and regulatory element activity. The vector construction was established by Shandong Jekaiyer Biotechnology Co., Ltd, Jinan, China (Supplementary Tables 6, 7).

### Proteomics technology

Proteins were extracted from tumors and tested by a BCA protein assay kit (GK5011). The peptide mixture was desalted by C18 ZipTip, quantified by Pierce™ Quantitative Colorimetric Peptide Assay (23275), and then lyophilized by SpeedVac. The phosphopeptides were selectively enriched with HiSelectTM TiO_2_ phosphopeptide Enrichment kit (Thermo Fisher Scientific, MA, USA) following the manufacturer’s instructions. DIA data was acquired in the diaPASEF mode. We defined 22 × 40 Th precursor isolation windows from *m*/*z* 349 to 1229. To adapt the MS1 cycle time, we set the repetitions to variable steps (2–5) in the 13-scan diaPASEF scheme in our experiment. During PASEF MSMS scanning, the collision energy was ramped linearly as a function of the mobility from 59 eV at 1/*K*0 = 1.6 V s/cm^2^ to 20 eV at 1/*K*0 = 0.6 V s/cm^2^. Tandem mass spectra were processed and analyzed by Spectronaut 18 (Biognosys AG, Switzerland) with default settings. Precursors that passed the 1% *Q*-value cutoff and contained phosphorylated sites were used to calculate the PTM site quantification [41].

### In vivo animal models

Nude mice (5 weeks old, female) were used to construct subcutaneous implant tumors (1 × 10^5^ cells in the right arm) and breast cancer liver metastasis tumors (1 × 10^4^ in the right liver) with MB-231, MCF-7^PPARD-KO^, 4T1, and 4T1-L cells, respectively. The breast cancer liver metastasis tumor was constructed in two ways: the cultured cancer cells were injected into the liver directly; the fresh subcutaneous tumors were cut into 1 mm^3^ and transplanted into the right liver lob. The parameters of subcutaneous tumors were recorded on the 7th day, with a volume estimated using the formula: tumor volume = 0.5 × length × width^2^ (mm^3^). The volume of liver tumors was measured by an ultra-high frequency color Doppler imaging system (FUJIVevo F2 LT, FUJIFLM VS company, USA) and Small Animal Imaging System (IVIS Lumina III, PerkinElmer, USA) after abdominal injecting with Luciferase potassium salt (C11H7KN2O3S2, PerkinElmer, Suzhou, China). Every mouse would be interfered with 5 μm DCA and 10 μm Rotenone (100 μL) in subcutaneous and abdominal cavity injections. After 21 days of subcutaneous tumor growth and 17 days of liver cancer cell injections, animals were sacrificed, and tumors were stored in a −80 °C refrigerator. All experimental procedures were conducted in accordance with the guidelines of the Laboratory Animal Center, Shandong provincial third hospital (SYDW-2023004). Efforts were made to minimize animal suffering.

### Statistical analysis

Differences between groups were analyzed by one-way or two-way ANOVA in SPSS 28.0, and the data were presented as the mean ± standard deviation (SD) with Student’s *t*-test. A *p*-value < 0.05 was regarded as statistically significant. The exact *p*-value was provided in the corresponding figure. *p* < 0.05(*), *p* < 0.01 (**), *p* < 0.001 (***) and *p* < 0.0001 (****) indicated statistically significant changes. For data presented without statistics, the experiment was repeated at least three times to ensure reproducibility.

## Supplementary information

original data- WB

supplement tables

## Data Availability

The raw sequence data reported in this paper have been deposited in the Genome Sequence Archive, China National Center for Bioinformation/Beijing Institute of Genomics, Chinese Academy of Sciences (GSA-Human: HRA011590; CRA017884) that are publicly accessible at https://ngdc.cncb.ac.cn.
