# Supplementary material for: Anaerobic metabolism promotes breast cancer survival via Histone-3 Lysine-18 lactylation mediating PPARD axis
Source: Cell Death Discov. 2025 Feb 8;11:54. doi: 10.1038/s41420-025-02334-x (PMC11807217; doi:10.1038/s41420-025-02334-x)
Supplement: Supplementary file 1 — original data- WB [file 41420_2025_2334_MOESM1_ESM.docx]

Raw images of Western blot

Figure 2-A:

| 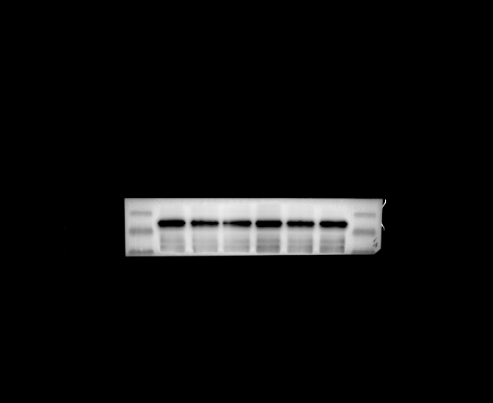  HDAC1 | 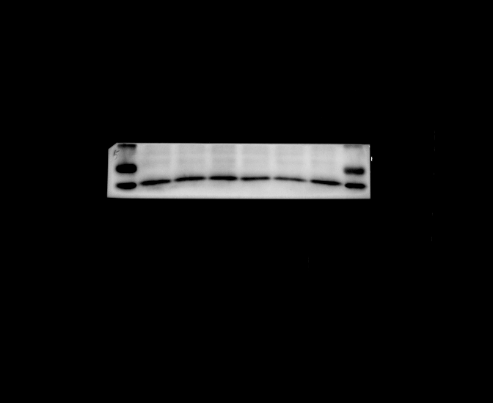  H3K18la |
| --- | --- |
| 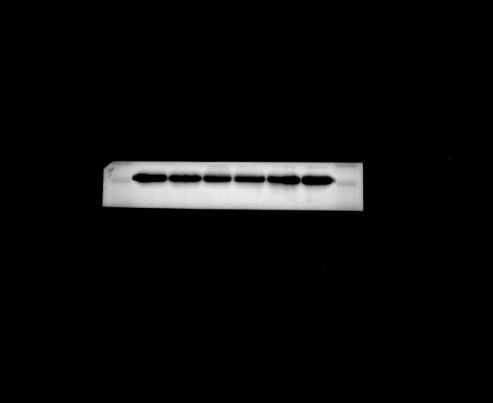  GAPDH | 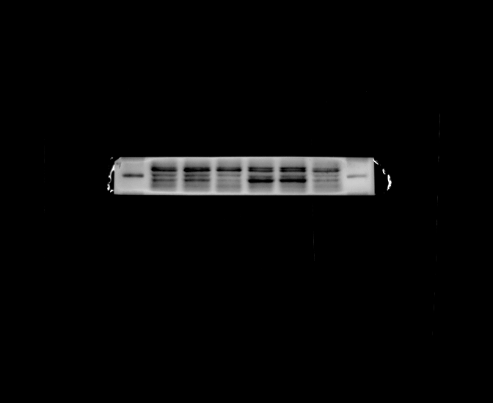  HDAC3 |
| 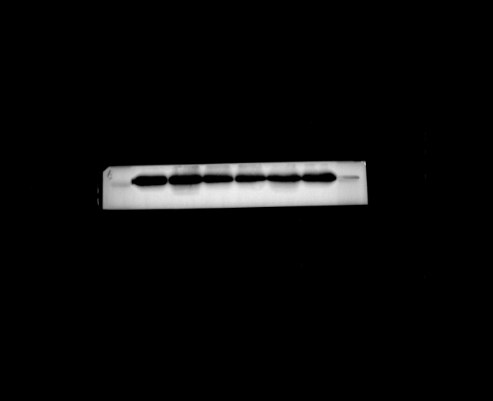  GAPDH | 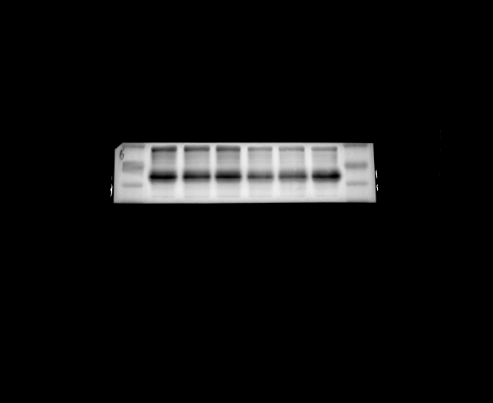  HDAC2 |
| 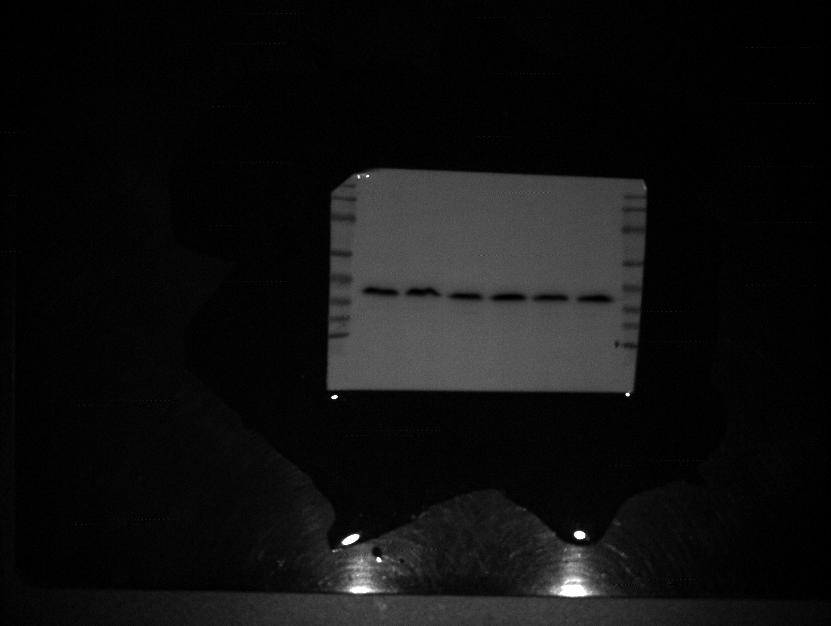GAPDH | |

Fig 2-D:

| H3K18la+GAPDH  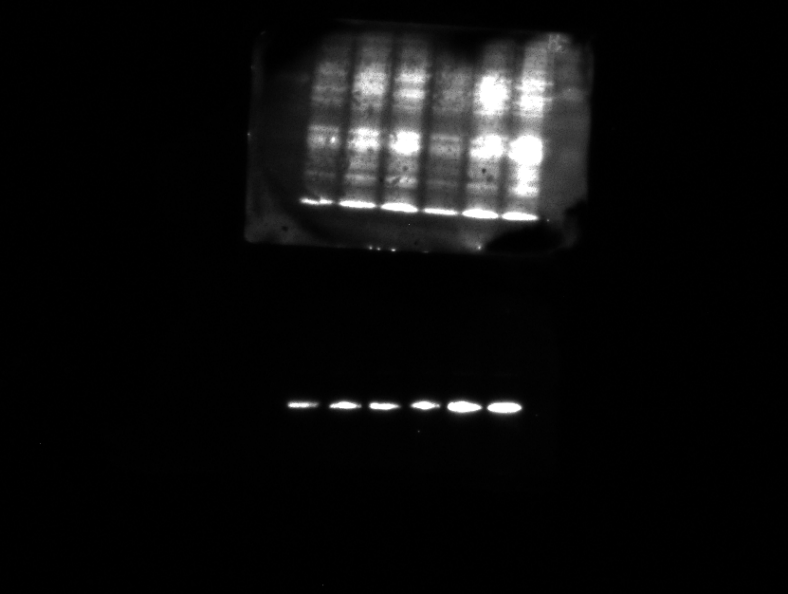  GAPDH  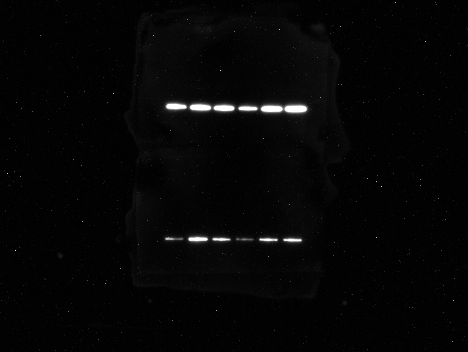 | HDAC1  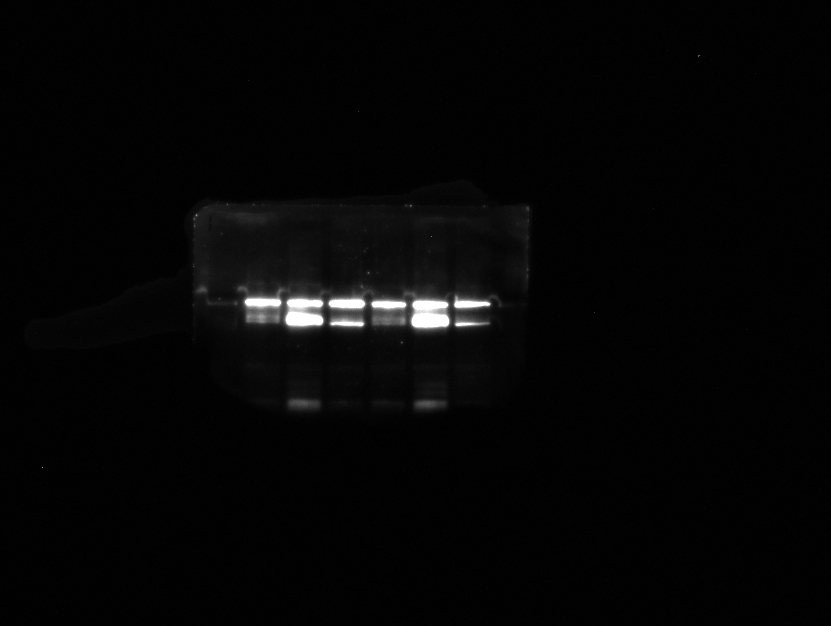  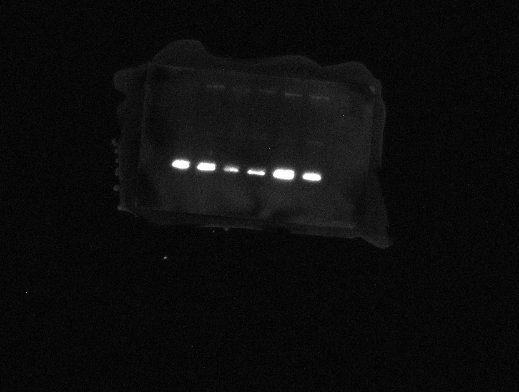HDAC2 |
| --- | --- |
| 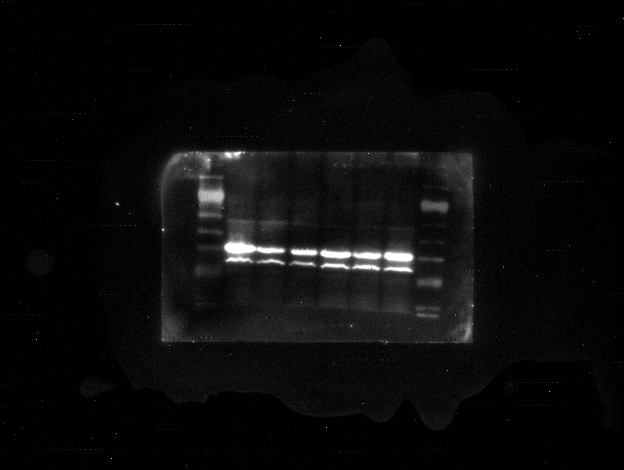HDAC3 | 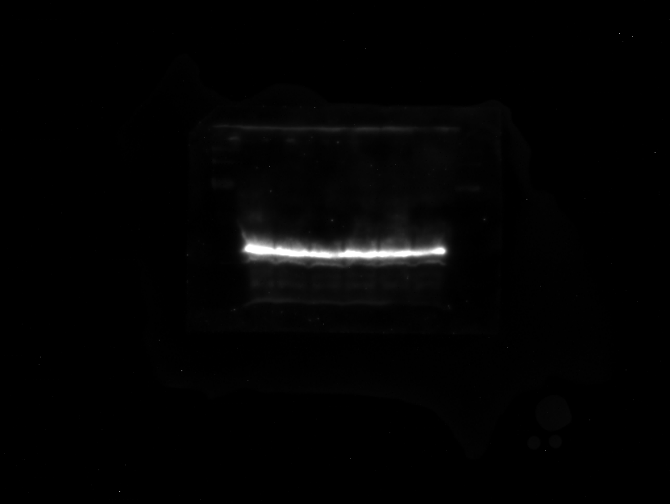GAPDH |

Fig 2-J， K:

| 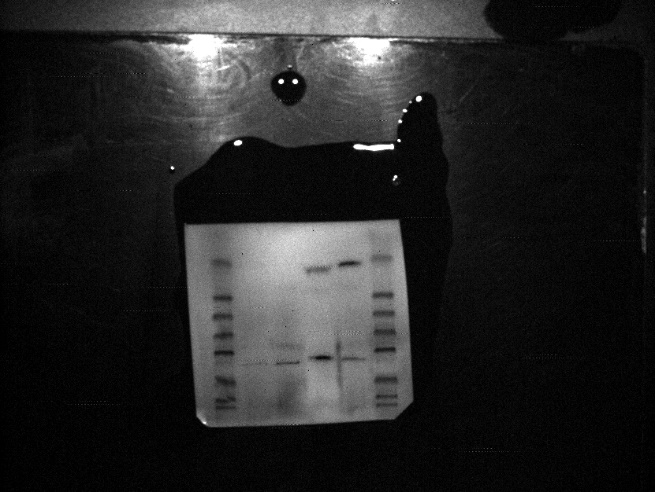HDAC3 | HDAC2  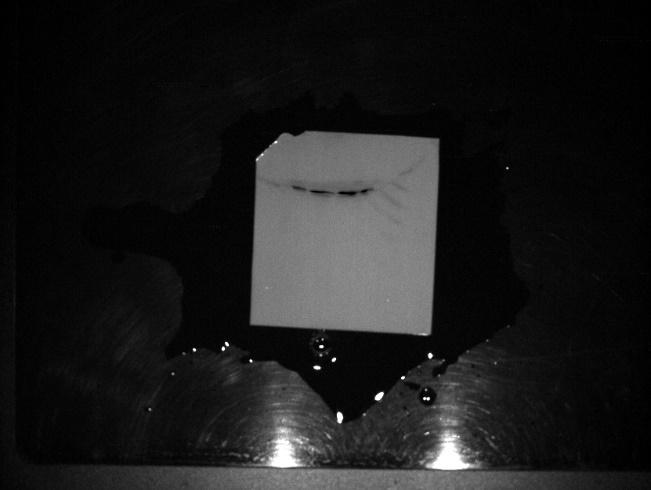 |
| --- | --- |
| 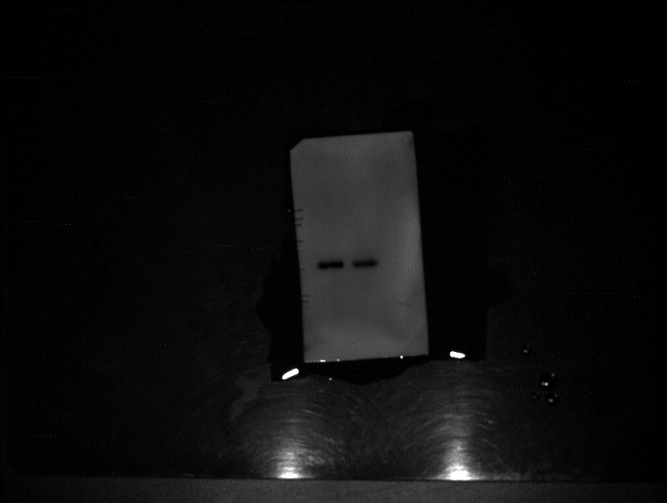GAPDH | 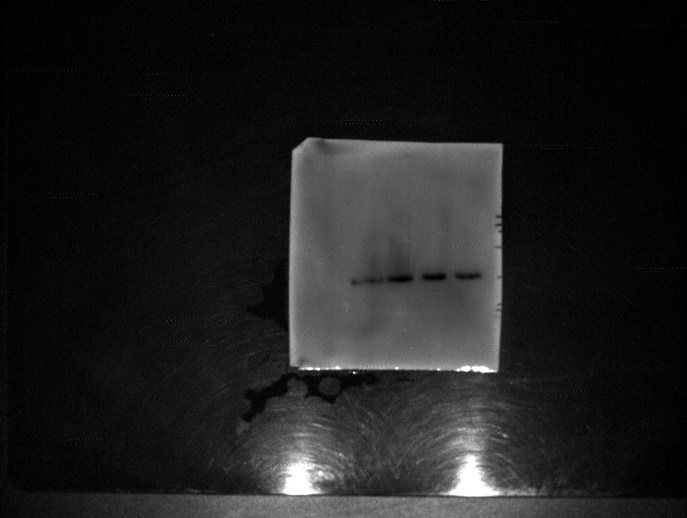H3K18la |
| GAPDH  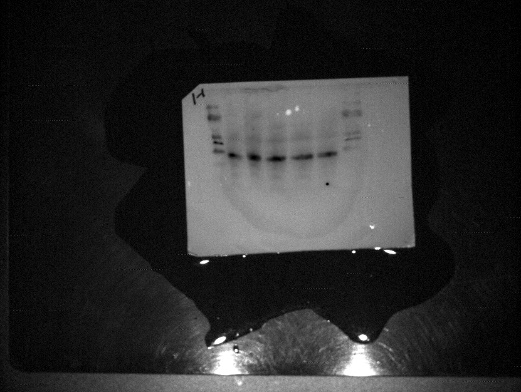 | |

Fig 3， I:

| 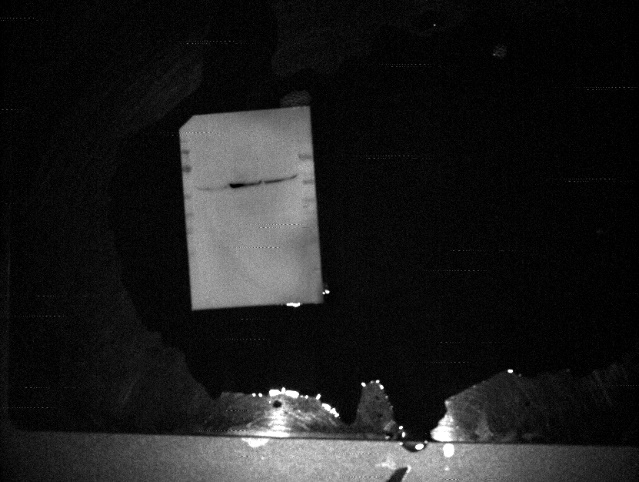PPARD | 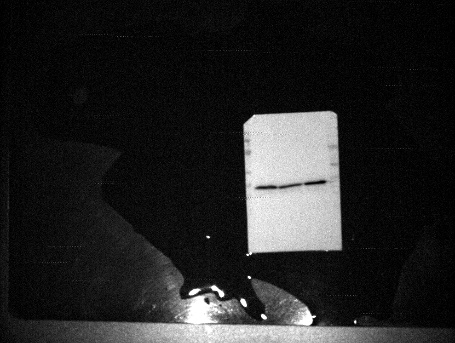GAPDH |
| --- | --- |

Fig 3， L:

| 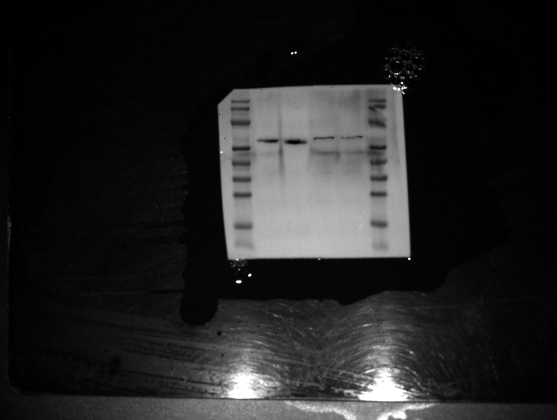PPARD | 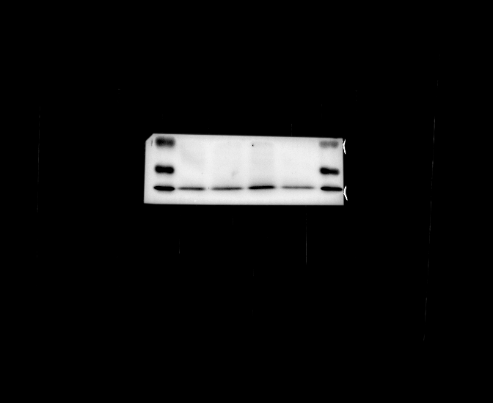H3K18la |
| --- | --- |
| 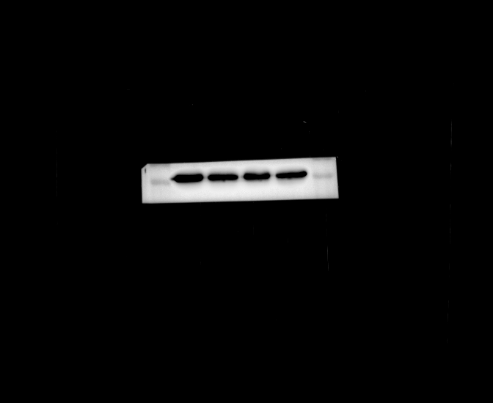GAPDH | |

Fig 4， L：

| 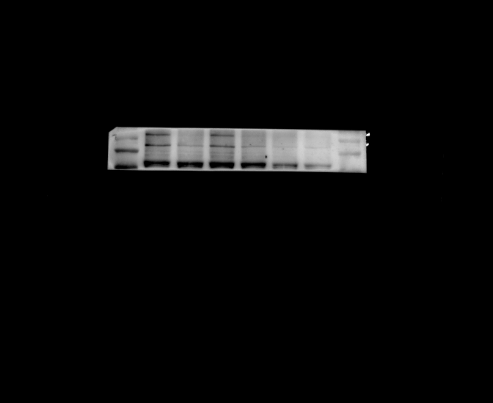PI3K | 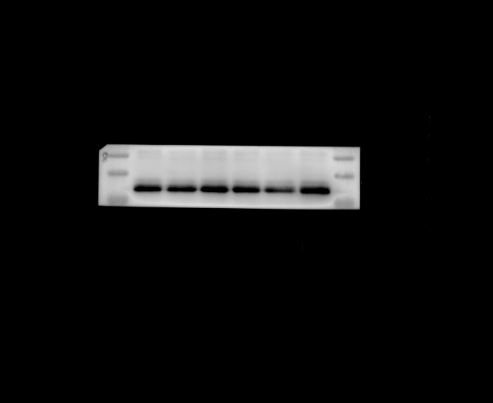CDK4 |
| --- | --- |
| 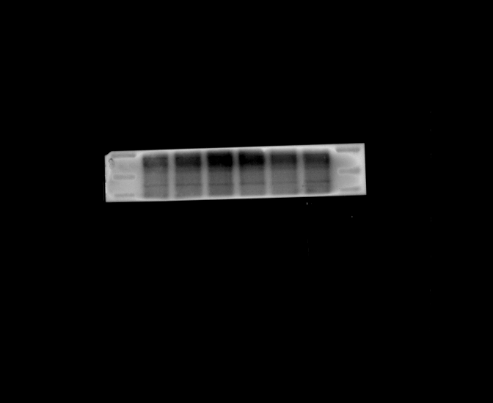  P-AKT | 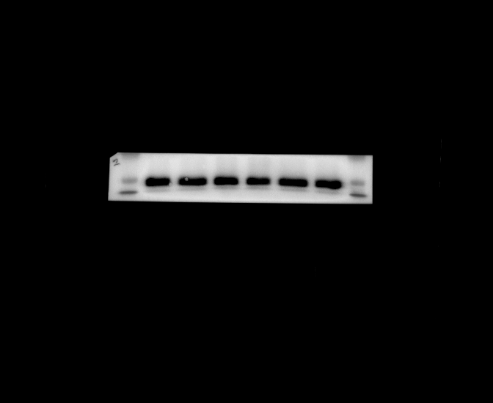BAX |
| 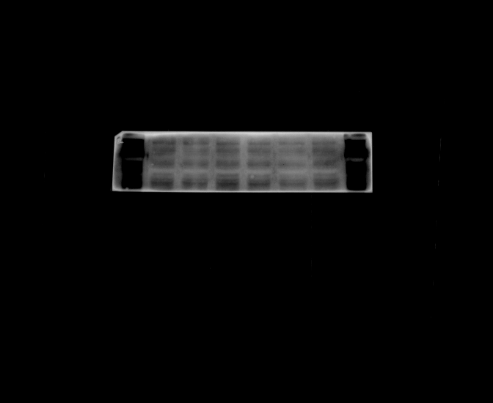ILK | 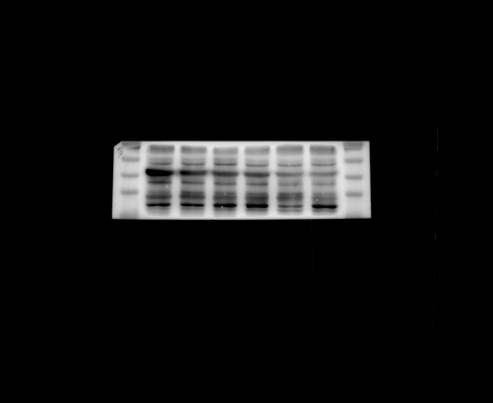CDK6 |
| 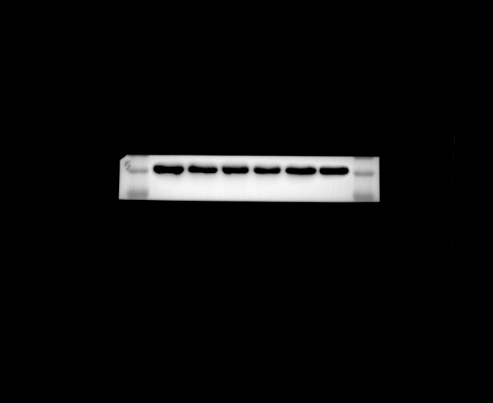GAPDH | 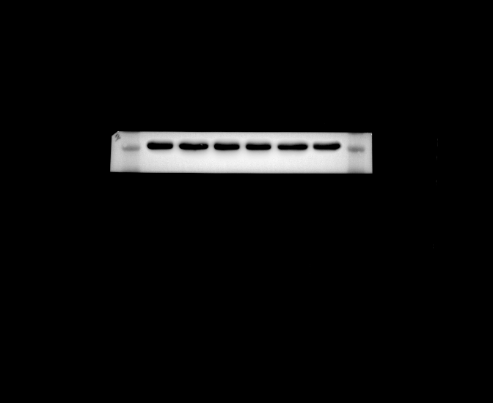GAPDH |
| 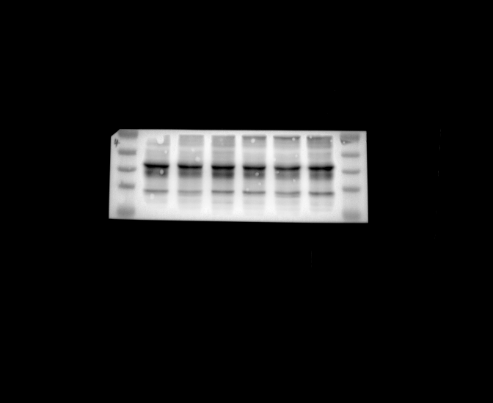Caspase-9 | |

Fig5，C:

| 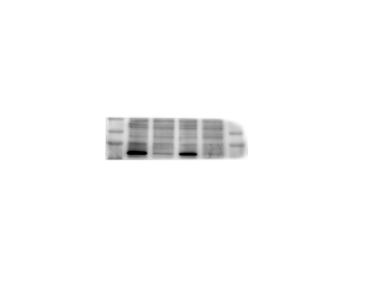CDK6 | 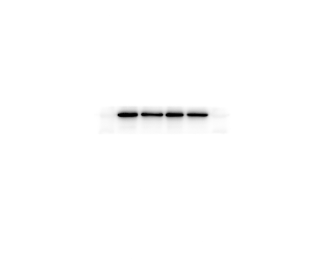GAPDH |
| --- | --- |
| 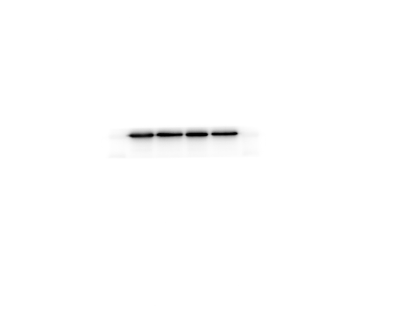GAPDH | 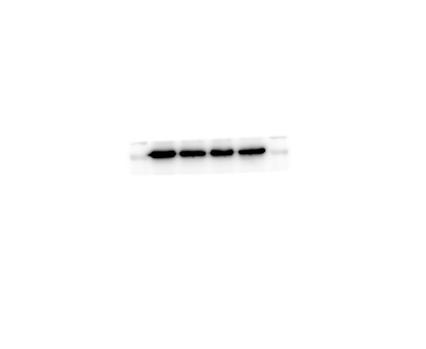GAPDH |
| 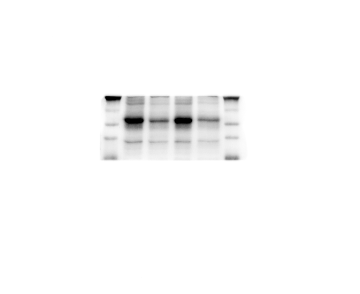Caspase-9 | 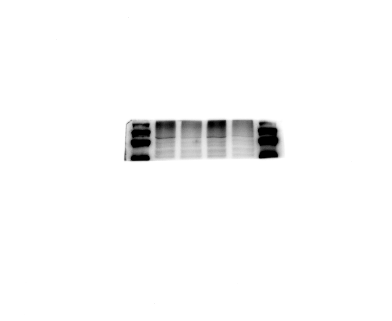PI3K |
| 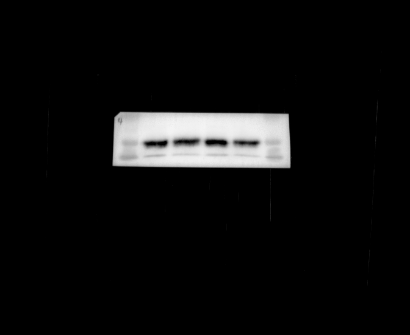BAX | 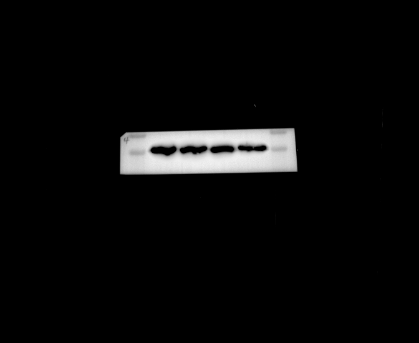GAPDH |
| 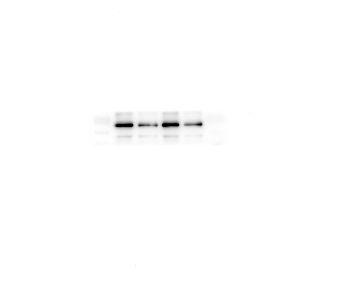P-AKT | 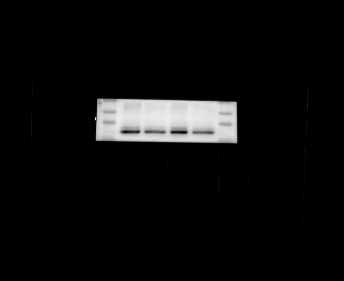CDK4 |

Fig 6，E, H:

| 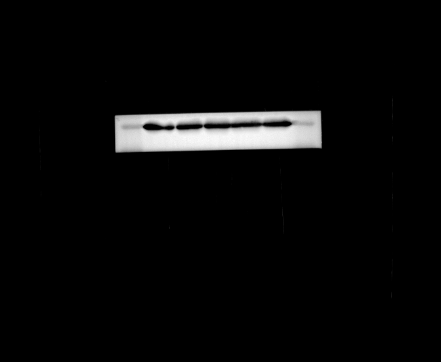GAPDH | 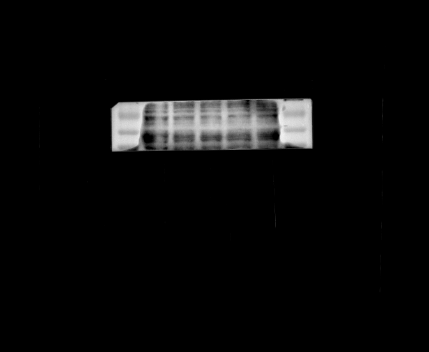ILK |
| --- | --- |
| 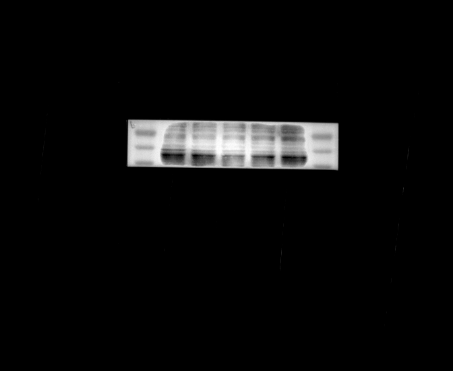ILK | 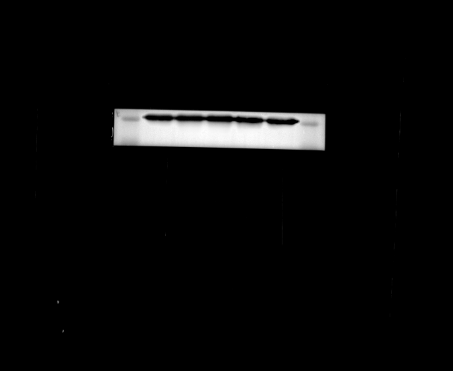GAPDH |
