# Supplementary material for: Anaerobic metabolism promotes breast cancer survival via Histone-3 Lysine-18 lactylation mediating PPARD axis
Source: Cell Death Discov. 2025 Feb 8;11:54. doi: 10.1038/s41420-025-02334-x (PMC11807217; doi:10.1038/s41420-025-02334-x)
Supplement: Supplementary file 2 — supplement tables [file 41420_2025_2334_MOESM2_ESM.docx]

| **Human Genes** | **F-sequences** | **R-sequences** |
| --- | --- | --- |
| HDAC1 | TGTCGGAGTACAGCAAGCAG | CACAGCACTTGCCACAGAAC |
| HDAC2 | AGCCACTGCCGAAGAAATGA | ACTGAACCGCCAGTTGAGAG |
| HDAC3 | AGGCCTCCCAACATGACATG | TGTGTAACGCGAGCAGAACT |
| AKT | GGACAAGGACGGGCACATTA | CGACCGCACATCATCTCGTA |
| ILK | GGAGGTACCCTTTGCTGACC | TCGCTTTGCAGGGTCTTCAT |
| PI3K | CAATCGGTGACTGTGTGGGA | CTGTTGAACTGCAGTGCACC |
| PPARD | GGCTTCCACTACGGTGTTCA | ACTTCTGGAAGCGGCAGTAC |
| Bax | TCTTTTTCCGAGTGGCAGCT | AGTCCAATGTCCAGCCCATG |
| Caspase9 | GCAAGCAGCAAAGTTGTCGA | TCAAGAGCACCGACATCACC |
| CDK4 | AGCTCCCGAAGTTCTTCTGC | CATCTCGAGGCCAGTCATCC |
| CDK6 | GTGTGCACAGTGTCACGAAC | AGATCGCGATGCACTACTCG |
| β-actin | CCACCATGTACCCTGGCATT | CGGACTCGTCATACTCCTGC |
|  |  |  |

Supplement Table 1. The qPCR sequences of human genes were listed.

| **Mouse Genes** | **F-sequences** | **R-sequences** |
| --- | --- | --- |
| H3K18la | TGGGTCTGTTTGAGGACACC | CCAGTTGGATGTCCTTGGGC |
| HDAC1 | CTGTGAACTACCCACTGCGA | TTGGCGTGTCCTTTGATGGT |
| HDAC2 | TGTCCGGTGTTTGATGGACT | ATTTCACAGCCCCAGCAACT |
| HDAC3 | TCAGCCCCACCAATATGCAG | TGTAACGGGAGCAGAACTCG |
| AKT | CCTCAAGAACGATGGCACCT | TGCAGGCAGCGGATGATAAA |
| ILK | GGACAACACAGAGAACGACCT | GATCAGCATTTCAACCACCGC |
| PI3K | GATCAGCATTTCAACCACCGC | ATGATGTTGCTGTTGTGCCG |
| PPARD | GCAGTGGCTAAAGAAGACGGA | CATGTCCTTGTAGATTTCCTGGAG |
| Bax | ACCAGGGTGGCTGGGAAG | CCTTTCCCCTTCCCCCATTC |
| Caspase9 | CGGTGGTGAGCAGAAAGACC | GGGGGTAGGCAAACTTGACA |
| CDK4 | GGAAACTCTGAAGCCGACCA | AGGTCAGCATTTCCAGTAGCA |
| CDK6 | CTTACCTCGGTGGTCGTCAC | AGATGCAACCGACACTCCAG |
| β-actin | GGCTGTATTCCCCTCCATCG | CCAGTTGGTAACAATGCCATGT |

Supplement Table 2. The qPCR sequences of mouse genes were listed.

| **Primary antibody** | **Company** | **Number** | **Dilution** |
| --- | --- | --- | --- |
| H3K18LA | PTMBio | PTM-1406RM | 1:3000 |
| HDAC1 | PTG | 66085-1-lg | 1:1000 |
| HDAC2 | PTG | 12922-3-AP | 1:1000 |
| HDAC3 | PTG | 10255-1-AP | 1:1000 |
| CDK4 | PTG | 11026-1-AP | 1:1000 |
| AKT | PTG | 10203-2-lg | 1:500 |
| PI3K | PTG | 67071-1-lg | 1:500 |
| CDK6 | PTG | 14052-1-AP | 1:1000 |
| YAP1 | PTG | 13584-1-AP | 1:1000 |
| ILK | PTG | 24926-1-AP | 1:1000 |

Supplement Table 3. The primary antibodies for IHC and IF were shown with company names and dilutions.

| **Primary antibody** | **Specie** | **Company** | **Number** | **Dillution** | **Weight** |
| --- | --- | --- | --- | --- | --- |
| HDAC1 | Mouse | Proteintech | 66085-1-lg | 1:5000 | 65kDa |
| CDK6 | Rabbit | Proteintech | 14052-1-AP | 1:2000 | 36-40kDa |
| HDAC2 | Rabbit | Proteintech | 12922-3-AP | 1:5000 | 55-60kDa |
| CDK4 | Rabbit | Proteintech | 11026-1-AP | 1:2000 | 34kDa |
| HDAC3 | Rabbit | Proteintech | 10255-1-AP | 1:1000 | 49kDa |
| PPARD | Rabbit | abcam | AB178866 | 1:1000 | 50kDa |
| H3K18Ia | Rabbit | PTMBIO | PTM-1406RM | 1:1000 | 15kDa |
| ILK | Rabbit | Proteintech | 12955-1-AP | 1:1000 | 51-59kDa |
| Caspase9 | Rabbit | Proteintech | 10380-1-AP | 1:1000 | 46,30-39kDa |
| PI3K | Mouse | Proteintech | 67071-1-Ig | 1:1000 | 110kDa |
| P-AKT | Rabbit | Proteintech | 80455-1-RR | 1:5000 | 58kDa |
| BAX | Rabbit | Proteintech | 50599-2-Ig | 1:2000 | 21kDa |
| ILK | Rabbit | Proteintech | 24926-1-AP | 1:1000 | 51-59kDa |
| GAPDH | Mouse | Proteintech | 60004-1-Ig | 1:5000 | 36kDa |

Supplement Table 4. The primary antibodies of western blot assay were shown.

| **Reagent** | **Concentration** | **Dilution** | **Total volume** |
| --- | --- | --- | --- |
| 1M CaCl2 | 30 mM | 33.33 | 3 mL |
| 1M Mg (Ac)2 | 18 mM | 55.56 | 1.8 mL |
| 1M Tris pH 7.8 | 60 mM | 16.67 | 6 mL |
| H2O |  |  | 89.2 mL |
| 6x Homogenization Buffer Stable | 6x | 1.00 | 648.84 ul |
| 100 mM PMSF | 0.1 mM | 1000.00 | 1.08 ul |
| 14.3 M β-mercaptoethanol | 1 mM | 14300.00 | 0.08 ul |
| 6x Homogenization Buffer Unstable | 1x | 6.00 | 333.33 ul |
| 1M Sucrose | 320 mM | 3.13 | 640.00 ul |
| 500 mM EDTA | 0.1 mM | 5000.00 | 0.40 ul |
| 10% NP40 | 0.1% | 100.00 | 20.00 ul |
| H2O |  |  | 1006.27 ul |

Supplement Table 5. The ATAC-seq reaction system were shown.

| **Vectors** | **Sequences** |
| --- | --- |
| pCDNA3.1-PPARD-F | CTTGGTACCGAGCTCGGATCCGCCACCATGGAGCAGCCACAGGAGG |
| pCDNA3.1-PPARD-R | ATGGTCTTTGTAGTCCTCGAGCTCACCTCCACACAGAATGATGG |
| pGL4.10-ILK-F | ctggcctaactggccggtaccATCACTCTTGTATGTAGAACCAGCTGA |
| pGL4.10-ILK-R | cagtaccggattgccaagcttGCCCGCAGCCCGCCGCCG |
| pGL4.10-AKT-F | TGAAGGAGTGACCGAGAT |
| pGL4.10-AKT-R | GATGGAGAAGGCAGGATG |

Supplement Table 6. The vector constructions and sequences were shown in Luciferase gene reports assay.

| **Gene Name** | **Sequence** |
| --- | --- |
| ILK-promoter-WT | AGATGTGGACCTGATTCAGACTGGCAGAAGCATCTGAATTCTTCCCAGTCAAGCCTGCCAGAACCCAGAAGAACTTTTATGTCACCACCCCAACTTCTGGGAAGCTGGAGAGCAGAGAAACCACCTATCCCACTTTCTTCAATGTGTAAATGAGCTAATGGAGGTTTCCCCAAAGGAAAATGCAAAGGCCACAAGCAACAAGACCAAGGCCAGAGACCAGGAATTCCAACCCTTCACCTTGAGCTTTTGCCTAGAGCTGATACATTTCTGTAAGAGGTAAGGAGGCTTCTGAACACAGGAGACACATGATCACACACGTATATTAAATATTTAACATGTCTCTAGCTGCAGTGTAGAAAACAAACTGAAGGGGAGAAATTGCAGACAGAGAGGCCAGCGGCTTGGACAATGGTAATGGGGCTAGGGGATGAAAAAGGAGGAAACAAAGCAGGCCGATTCCATGTTGGAGGCAGACTGAAGGGTAAGAAAAGCCTGAGGAAGCAGGGCCCTCACATCTGCAGAGGCTCAAAGGAGAAGATGTATTGTCGGGAAGGCTGATTCTGTGTTGCAGTCCTACCAGGTAATCCTAGGCCAGGCACTTACCCTGATTCTAAGTTTCCCACACCCTCCTTTGAACAATGATACTGACAACTCTTACTGTCTCCAATCCATCCTCCACACTGTAACCGACGACCCTTTCAAAATACAAATCTGAAGAAATAATTCCATTGCTTAAAACCTTTCATTGGCTTCTATTCTCAATATACGCAAAA |
| ILK-promoter-MUT | AGATGTGGACCTGATTCAGACTGGCAGAAGCATCTGAATTCTTCCCAGTCAAGCCTGCCAGAACCCAGAAGAACTTTTATGTCACCACCCCAACTTCTGGGAAGCTGGAGAGCAGAGAAACCACCTATCCCACTTTCTTCAATGTGTAAATGAGCTAATGGAGGTTTCCCCAAAGGAAAATGCAAAGGCCACAAGCAACAAGACCAAGGCCAGAGACCAGGAATTCCAACAACCAACCTTGAGCTTTTGCCTAGAGCTGATACATTTCTGTAAGAGGTAAGGAGGCTTCTGAACACAGGAGACACATGATCACACACGTATATTAAATATTTAACATGTCTCTAGCTGCAGTGTAGAAAACAAACTGAAGGGGAGAAATTGCAGACAGAGAGGCCAGCGGCTTGGACAATGGTAATGGGGCTAGGGGATGAAAAAGGAGGAAACAAAGCAGGCCGATTCCATGTTGGAGGCAGACTGAAGGGTAAGAAAAGCCTGAGGAAGCAGGGCCCTCACATCTGCAGAGGCTCAAAGGAGAAGATGTATTGTCGGGAAGGCTGATTCTGTGTTGCAGTCCTACCAGGTAATCCTAGGCCAGGCACT |
| AKT-promoter-WT | GGCGGCGGCAGGACCGAGCGCGGCAGGCGGCTGGCCCAGCGCACGCAGCGCGGCCCGAAGACGGGAGCAGGCGGCCGAGCACCGAGCGCTGGGCACCGGGCACCGAGCGGCGGCGGCACGCGAGGCCCGGCCCCGAGCAGCGCCCCCGCCCGCCGCGGCCTCCAGCCCGGCCCCGCCCAGCGCCGGCCCGCGGGATGCGGAGCGGCGGGCGCCGGAGGCCGCGGCCCGGCTAGGCCCGCGCTCGCGCCCGGACGCGGCGGCCCGGTGAGTCCCCGCCCGCCGTGGCCGCCCGGGCCTGGATTTCCTCCCCGCGGGCCGGGCCGCTTTGTTCGCGGCCGGTCGGGCCGGGGCGCGAGCCGCGGCGCCGCCAGAATGGAGGAGCGGGAGCAGGAAGTGGCCGAGCGGGCCTGGGCGGGGAGGGCGCGGGGCGCGCGGGCCCGGCCAAGGGAGGGCGGCCCCACGCCGGGCGCCGGGGGTGCAGGCTGCCGGCCCCAGCCTCCCTCATGACCTTG |
| AKT-promoter-MUT | GGCGGCGGCAGGACCGAGCGCGGCAGGCGGCTGGCCCAGCGCACGCAGCGCGGCCCGAAGACGGGAGCAGGCGGCCGAGCACCGAGCGCTGGGCACCGGGCACCGAGCGGCGGCGGCACGCGAGGCCCGGCCCCGAGCAGCGCCCCCGCCCGCCGCGGCCTCCAGCCCGGCCCCGCCCAGCGCCGGCCCGCGGGATGCGGAGCGGCGGGCGCCGGAGGCCGCGGCCCGGCTAGGCCCGCGCTCGCGCCCGGACGCGGCGGCCCGGTGAGTCCCCGCCCGCCGTGGCCGCCCGGGCCTGGATTTCCTCCGGCGCCCGGCCCGGCCTTTGTTCGCGGCCGGTCGGGCCGGGGCGCGAGCCGCGGCGCCGCCAGAATGGAGGAGCGGGAGCAGGAAGTGGCCGAGCGGGCCTGGGCGGGGAGGGCGCGGGGCGCGCGGGCCCGGCCAAGGGAGGGCGGCCCCACGCCGGGCGCCGGGGGTGCAGGCTGCCGGCCCCAGCCTCCCTCATGACCTTGGGGAGGCCGCTCCGCCGGGCGAGG |

Supplement Table 7. The wild and mutant gene sequences used in the Luciferase gene reports assay were shown. Please note that partial sequences were deleted because of the limitation of tables.

| Ontology | ID | Description | Gene Ratio | *P* value | *p*.adjust |
| --- | --- | --- | --- | --- | --- |
| BP | GO:0045927 | positive regulation of growth | 16/34 | 1.54e-21 | 3.35e-18 |
| BP | GO:0071453 | cellular response to oxygen levels | 13/34 | 9.73e-19 | 8.92e-16 |
| BP | GO:0071322 | cellular response to carbohydrate stimulus | 13/34 | 1.23e-18 | 8.92e-16 |
| BP | GO:0036294 | cellular response to decreased oxygen levels | 15/34 | 3.51e-18 | 1.9e-15 |
| BP | GO:0071456 | cellular response to hypoxia | 12/34 | 1.11e-17 | 4.83e-15 |
| BP | GO:0071333 | cellular response to glucose stimulus | 10/34 | 0.00560098 | 0.04804219 |
| CC | GO:0005667 | transcription regulator complex | 12/34 | 1.47e-11 | 3.98e-10 |
| CC | GO:0090575 | RNA polymerase II transcription regulator complex | 9/34 | 1.47e-10 | 1.98e-09 |
| CC | GO:0090571 | RNA polymerase II transcription repressor complex | 2/34 | 0.0003 | 0.0024 |
| CC | GO:0000791 | euchromatin | 2/34 | 0.0052 | 0.0350 |
| CC | GO:0017053 | transcription repressor complex | 2/34 | 0.0077 | 0.0415 |
| MF | GO:0001221 | transcription coregulator binding | 12/34 | 4.36e-19 | 1.78e-17 |
| MF | GO:0001228 | DNA-binding transcription activator activity, RNA polymerase II-specific | 17/34 | 7.31e-19 | 1.78e-17 |
| MF | GO:0001216 | DNA-binding transcription activator activity | 17/34 | 8.46e-19 | 1.78e-17 |
| MF | GO:0140297 | DNA-binding transcription factor binding | 17/34 | 9.77e-19 | 1.78e-17 |
| MF | GO:0061629 | RNA polymerase II-specific DNA-binding transcription factor binding | 15/34 | 1.4e-17 | 2.04e-16 |
| KEGG | hsa04211 | Longevity regulating pathway | 4/21 | 6.85e-05 | 0.0060 |
| KEGG | hsa04659 | Th17 cell differentiation | 4/21 | 0.0001 | 0.0060 |
| KEGG | hsa05166 | Human T-cell leukemia virus 1 infection | 5/21 | 0.0002 | 0.0060 |
| KEGG | hsa04152 | AMPK signaling pathway | 4/21 | 0.0002 | 0.0060 |
| KEGG | hsa04218 | Cellular senescence | 4/21 | 0.0006 | 0.0105 |

Supplement Table 8. The GO/KEGG analysis of co-transcript factors in breast cancer and para-cancer tissues, involving in cell growth and oxygen metabolism.

| Gene Name | Foldchange | *P* value |
| --- | --- | --- |
| PTPN4 | 6.702052642 | 6.80E-78 |
| CPPED1 | 5.473430875 | 3.27E-69 |
| PPARD | 4.717124159 | 3.51E-36 |
| CCDC88A | 4.517151248 | 1.70E-106 |
| GRID2 | 4.456563887 | 1.01E-83 |
| ODF2L | 4.215668542 | 9.25E-75 |
| DENND1B | 4.135656874 | 1.67E-75 |
| NADK2 | 3.990793331 | 3.66E-35 |
| FEM1C | 3.923152766 | 3.52E-75 |
| PLSCR1 | 3.887270271 | 1.21E-89 |

Supplement Table 9. The top 10 downregulated genes of H3K18la with Fold-enrichment and *P* value by ChIP-sequence assay.

**Supplement Figures**


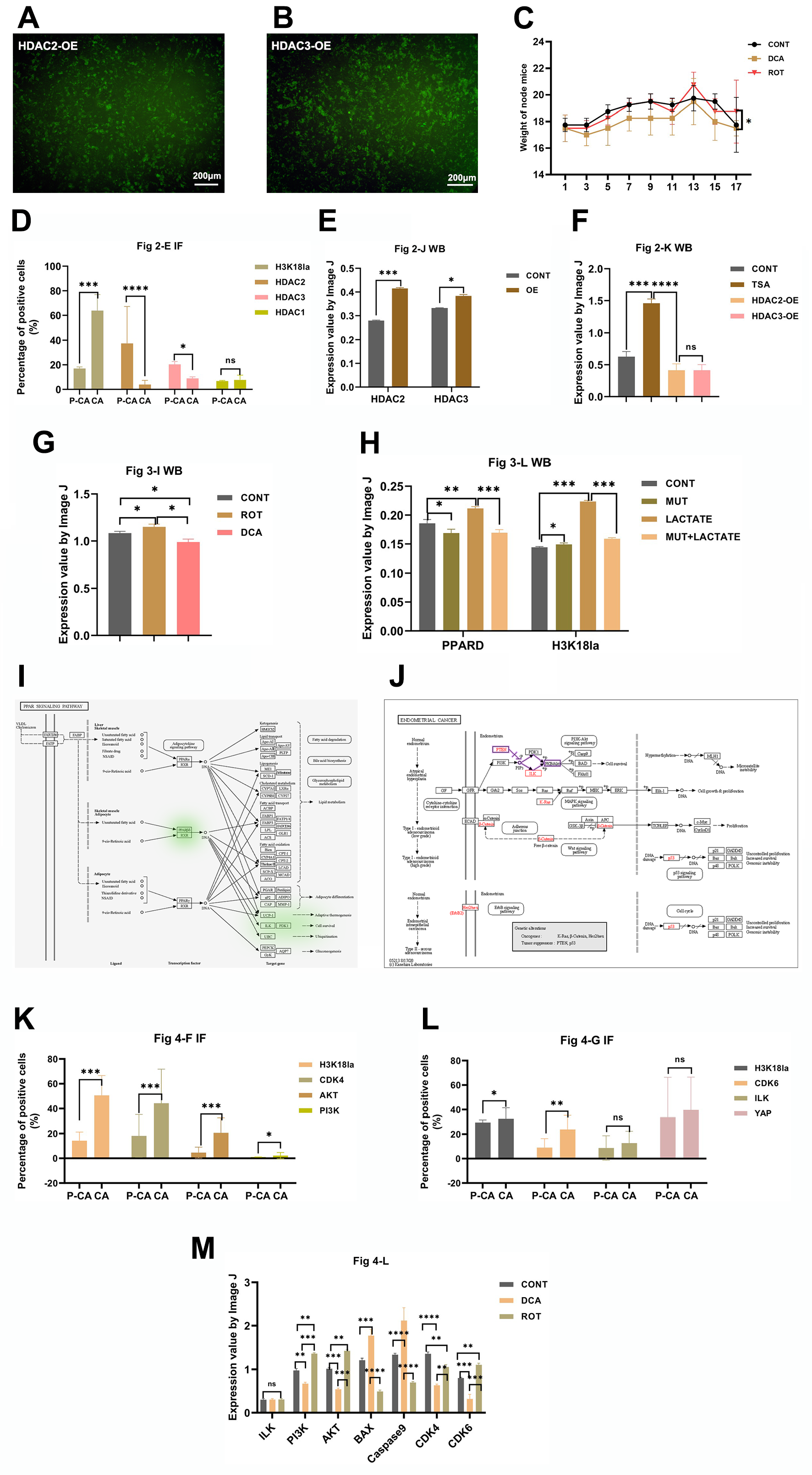


**Supplement Figure 1. Additional data performances.** A-B：The transfection fluorescence intensity of HDAC2 and HDAC3 overexpression plasmid treated in the MB-231 cells. C: The weight changes of breast cancer cell liver metastatic model of node mice. D-H: Associated quantifications were measured by Image J software. I-J: KEGG data analyzed PPARD and ILK associated signaling pathways. K-M: Associated quantifications were measured by Image J software.Data are mean ± SD. **P* < 0.05, ***P* ≤ 0.01, ****P* < 0.001, *****P* ≤ 0.0001.


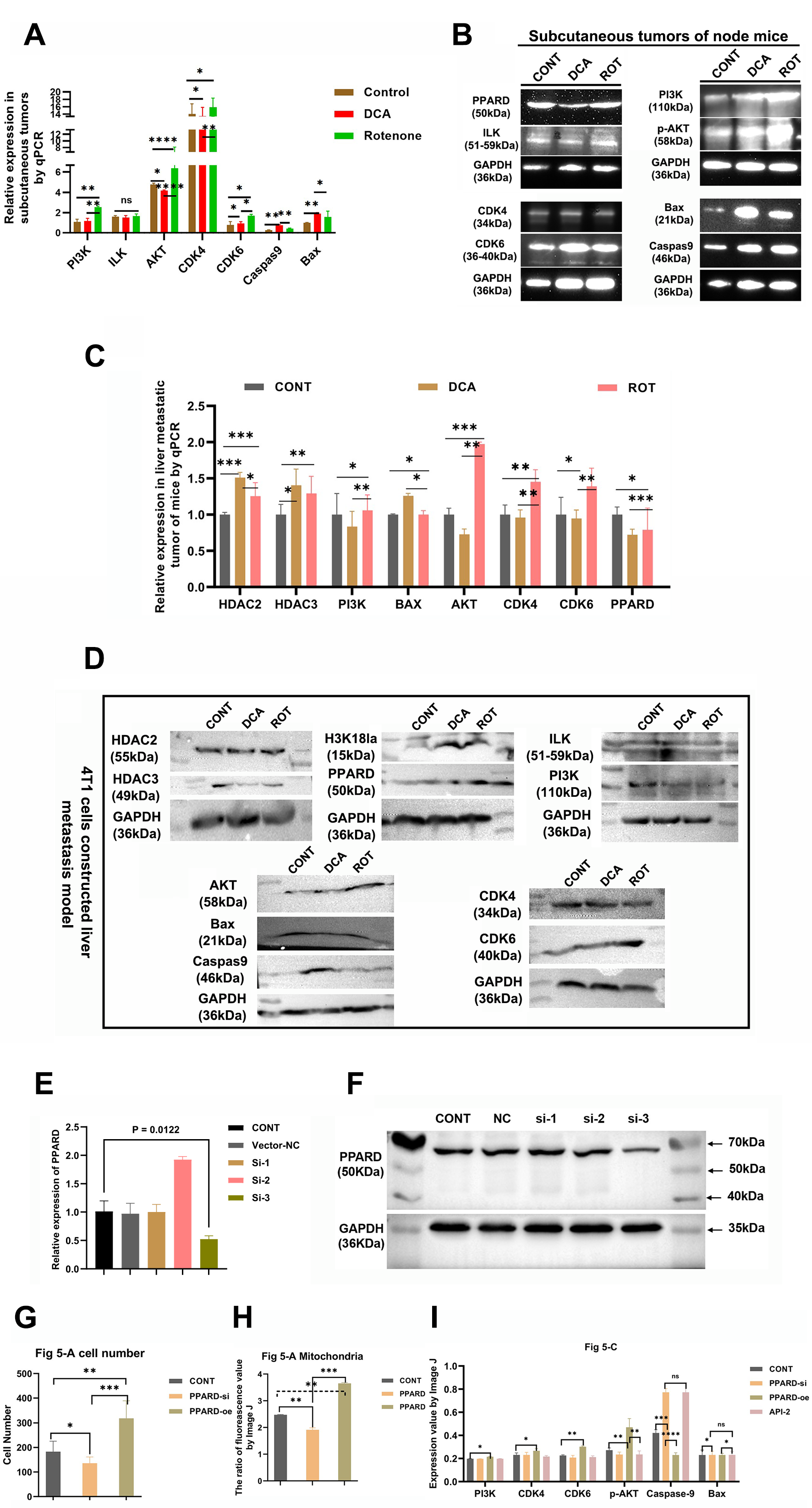


**Supplement Figure 2. Additional experiments data performances.** A-B: AKT signaling pathway associated marks were detected by qPCR and Western blot assays in subcutaneous tumors of node mice treated with DCA and Rotenone. C-D: AKT signaling pathway associated marks, HDACs family, H3K18la and PPARD were detected by qPCR and Western blot assays in breast cancer cell (4T1) liver metastasis tumors of node mice treated with DCA and Rotenone. E-F: PPARD expressions were verified in three silence RNA plasmid treated in MB-231 cells by qPCR and Western blot assays. G-I: Associated quantifications were measured by Image J software. Data are mean ± SD. **P* < 0.05, ***P* ≤ 0.01, ****P* < 0.001, *****P* ≤ 0.0001.


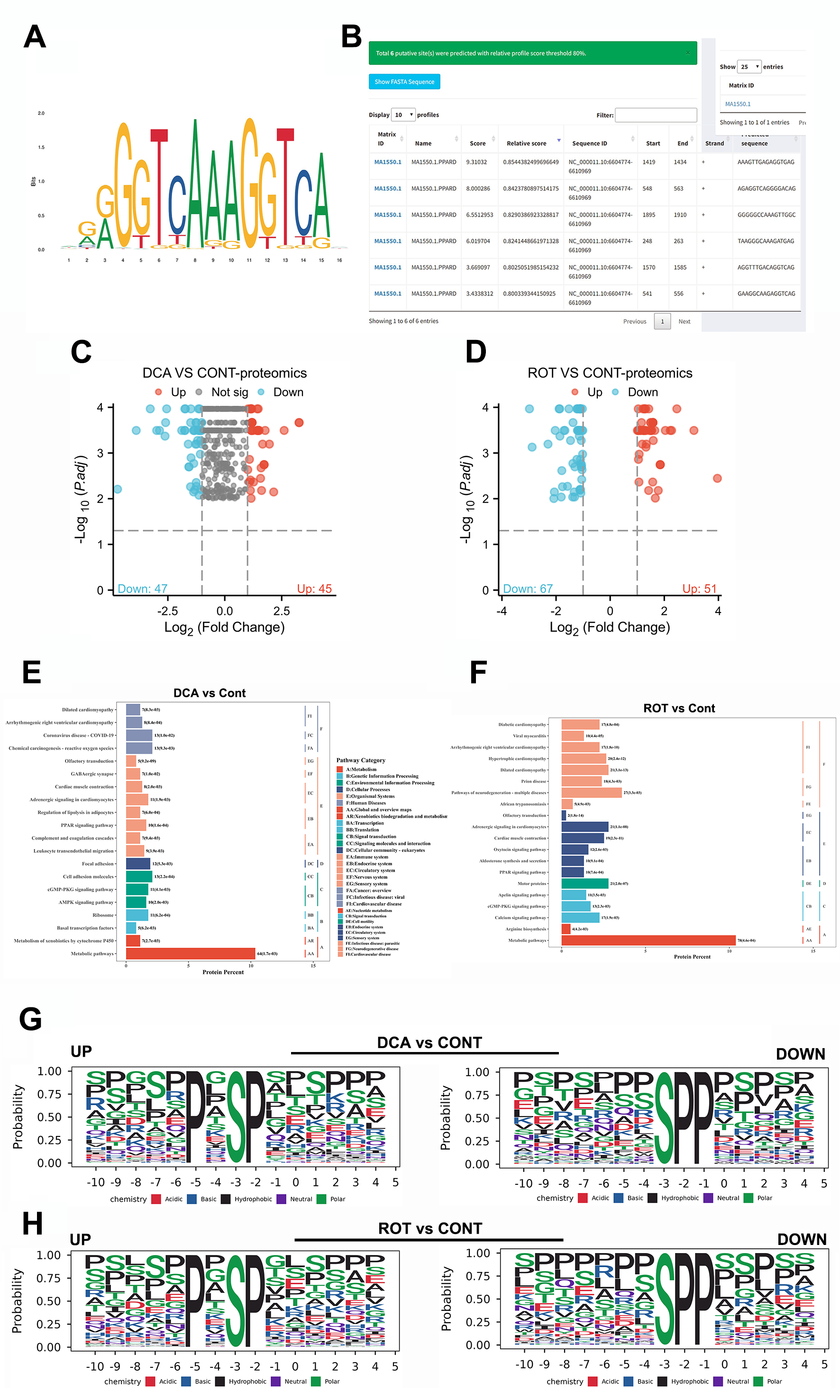


**Supplement Figure 3. Bioinformatics analysis of phosphorylation protein.** A-B: The predictive binding sites of PPARD as a transfactor based on the JASPA database. C-D: The volcano diagrams of DCA group and Rotenone group compared to the control group with subcutaneous tumors of node mice. E-F: KEGG pathway analysis of the differentially phosphorylated proteins in DCA and Rotenone groups. G-H: The most upregulated and downregulated motif sequences in the DCA versus control, and Rotenone versus control comparisons.

**List of abbreviations**

 BC - Breast Cancer

 PPARD - Peroxisome Proliferator-Activated Receptor Delta

 AKT - Protein Kinase B (often referred to as AKT)

 ILK - Integrin-Linked Kinase

 ATAC-seq - Assay for Transposase-Accessible Chromatin with high-throughput sequencing

 HDAC2 - Histone Deacetylase 2

 HDAC3 - Histone Deacetylase 3

 H3K18la - Histone 3 Lysine 18 Lactylation

 DMEM - Dulbecco's Modified Eagle Medium

 DCA - Dichloroacetic Acid

 DMSO - Dimethyl Sulfoxide

 PCR - Polymerase Chain Reaction

 ChIP - Chromatin Immunoprecipitation

IHC - Immunohistochemistry

 IF - Immunofluorescence

 HE - Hematein-Eosin

 ChIP-seq - Chromatin Immunoprecipitation Sequencing

 GO - Gene Ontology

 KEGG - Kyoto Encyclopedia of Genes and Genomes

 TCGA - The Cancer Genome Atlas

 PI - Propidium Iodide

 FITC - Fluorescein Isothiocyanate

 GFP - Green Fluorescent Protein

 Tn5 - Transposase Enzyme Tn5

 MS - Mass Spectrometry

 PVDF - Polyvinylidene Difluoride

 HRP - Horseradish Peroxidase

 BCA - Bicinchoninic Acid
